# Halogen-Bonding-Mediated Radical Reactions: The Unexpected Behavior of Piperazine-Based Dithiooxamide Ligands in the Presence of Diiodine

**DOI:** 10.1021/acs.inorgchem.2c02340

**Published:** 2023-01-05

**Authors:** Silvia Rizzato, Gabriele Manca, Marie-Hélène Lemée, Luciano Marchiò, Flaminia Cesare Marincola, Annalisa Guerri, Andrea Ienco, Angela Serpe, Paola Deplano

**Affiliations:** † Dipartimento di Chimica, Università degli Studi di Milano, Via Golgi 19, I-20133 Milano, Italy; ‡ Istituto di Chimica dei Composti Organometallici ICCOM-CNR, Via Madonna del Piano 10, I-50019 Sesto Fiorentino, Florence, Italy; § Institut Laue-Langevin, 71 avenue des Martyrs, CS 20156, 38042 Grenoble Cedex 9, France; ∥ Dipartimento di Chimica, Scienze della Vita e della Sostenibilità Ambientale, Università di Parma, 43124 Parma, Italy; ⊥ Dipartimento di Scienze Chimiche e Geologiche, Università di Cagliari, 09042 Monserrato, Cagliari, Italy; # Dipartimento di Chimica “Ugo Schiff”, Università di Firenze, Via della Lastruccia 3, 50019 Sesto Fiorentino, Firenze, Italy; ∇ Dipartimento di Ingegneria Civile, Ambientale e Architettura (DICAAR) and Research Unit of INSTM, Università di Cagliari, I-09042 Monserrato, Cagliari, Italy; ○ Istituto di Geologia Ambientale e Geoingegneria del Consiglio Nazionale delle Ricerche (IGAG-CNR), Piazza d’Armi, 09123 Cagliari, Italy

## Abstract

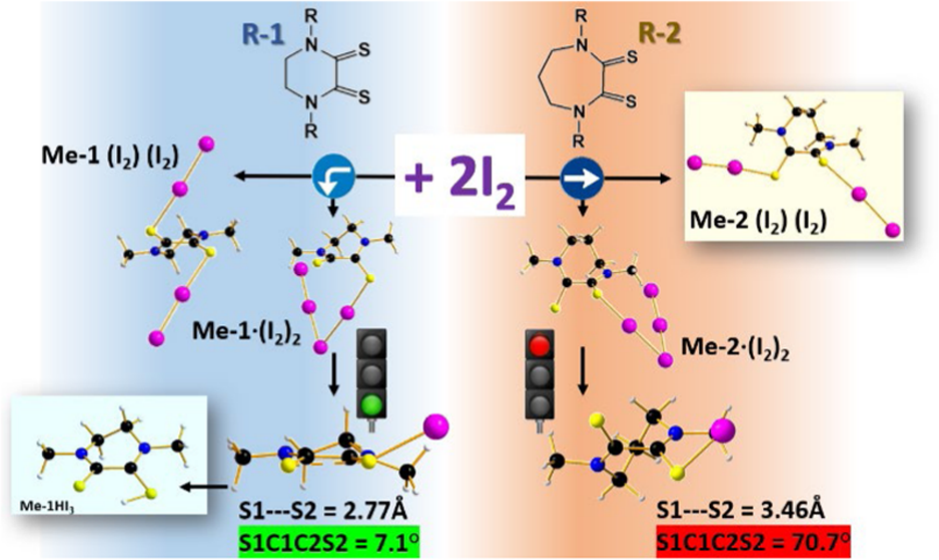

*N*,*N*′-Dialkylpiperazine-2,3-dithiones (R_2_pipdt) were recognized as a class of hexa-atomic cyclic dithiooxamide ligands with peculiar charge-transfer donor properties toward soft electron-acceptors such as noble metal cations and diiodine. The latter interaction is nowadays better described as halogen bonding. In the reaction with diiodine, R_2_pipdt unexpectedly provides the corresponding triiodide salts, differently from the other dithiooxamides, which instead typically achieve ligand·nI_2_ halogen-bonded adducts. In this paper, we report a combined experimental and theoretical study that allows elucidation of the nature of the cited products and the reasons behind the unpredictable behavior of these ligands. Specifically, low-temperature single-crystal X-ray diffraction measurements on a series of synthetically obtained R_2_pipdt (R = Me, *^i^*Pr, Bz)/I_3_ salts, complemented by neutron diffraction experiments, were able to experimentally highlight the formation of [R_2_pipdtH]^+^ cations with a −S–H bond on the dithionic moiety. Differently, with R = Ph, a benzothiazolylium cation, resulting from an intramolecular condensation reaction of the ligand, is obtained. Based on density functional theory (DFT) calculations, a reasonable reaction mechanism where diiodine plays the fundamental role of promoting a halogen-bonding-mediated radical reaction has been proposed. In addition, the comparison of combined experimental and computational results with the corresponding reactions of *N*,*N*′-dialkylperhydrodiazepine-2,3-dithione (R_2_dazdt, a hepta-atomic cyclic dithiooxamide), which provide neutral halogen-bonded adducts, pointed out that the difference in the torsion angle of the free ligands represents the structural key factor in determining the different reactivities of the two systems.

## Abstract 2

The I_3_^−^ salt of the S-protonated ligand has been established as the main product of the reaction of R_2_pipdt (**R-1**, R = Me, *^i^*Pr, Bz,···) with diiodine in organic solvents. The S−C−C−S low torsion angle was demonstrated to be the key structural factor affecting the overall reactivity. DFT calculations allowed us to speculate on a reasonable reaction mechanism mediated by the three-center-four-electron S−S−I bond formation followed by radical activation.

## 1 Introduction

**Chart 1:**
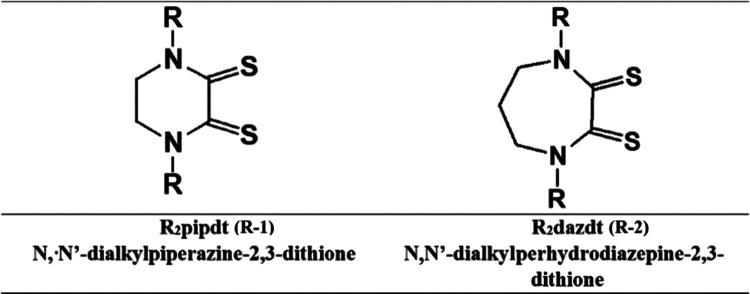
Cyclic Dithiooxamide Ligands of This Work

Dithiooxamides are well-known organic ligands largely employed in coordination chemistry for a long time thanks to the peculiar electronic properties and coordination versatility of the two vicinal thioamidic moieties.^1−4^ Despite the variety of interactions they can give rise to with metallic and nonmetallic species, dithiooxamides typically work as soft S-donors toward acceptors such as soft metal cations and halogens.^5−7^ The acceptor capability of the electron density of halogens from a neutral or anionic Lewis base is well recognized as a noncovalent attractive interaction involving a halogen as an acceptor to form adducts.^8^ This capability has also been elucidated by quantum mechanical methods giving rise to the widely accepted σ-hole model.^9,10^ In several cases, these adducts are stable in solution and can be isolated also in the solid state, while elsewhere the system further evolves to give redox products. The driving force of further evolution of the adduct is related to the σ electronic density distribution over the three centers S–I–I and also to the stability of the formed products.^11^ Numerous examples of organic substrates forming halogen-bonded adducts with iodine through their thiocarbonyl groups are known.^12−15^ Among them, *N*,*N*′-dialkylperhydrodiazepine-2,3-dithione (R_2_dazdt; Chart 1), a class of hepta-atomic cyclic dithiooxamides, provides 1:1 and 1:2 diiodine halogen-bonded adducts, similarly to those obtained with other acyclic dithiooxamide donors.^8^

These adducts, which have been isolated at the solid state and structurally characterized, behave as powerful lixiviants toward noble metals (Cu, Pd, Au) but are not effective toward platinum.^8,16,17^ The use of these lixiviants for noble metal leaching and recovery purposes has been extensively explored for smart applications through a green chemistry approach.^17−21^ On the other side, unexpected different behavior in the reaction with iodine was found for *N*,*N*′-dialkylpiperazine-2,3-dithione (R_2_pipdt; Chart 1), a corresponding class of cyclic dithiooxamides where the thioamide moieties are embedded in a hexa-atomic heterocycle.^8^ Indeed, the reaction of R_2_pipdt (R = Me) with I_2_ did not provide the expected halogen-bonded adduct as the final product. The X-ray diffraction (XRD) measurements at room temperature of the isolated product, dating back to the late nineties, in agreement with other analytical and spectroscopic (UV–vis–infrared (IR) and Raman) characterization, suggested the formation of the [Me_2_pipdt]I_3_, the triiodide salt of the ligand in the form of a cation. Based on the highly symmetrical configuration found for the ligand moiety by XRD (refined in the **C**2/**c** spatial group) as well as the absence of any diagnostic evidence of protonation by the other techniques, the most reasonable description of the system involved the formation of the radical [Me_2_pipdt]^•+^. However, no experimental support was found to demonstrate the radical formation. This reopened the discussion on a possible protonation of the ligand.^8^ Assumed this latter hypothesis, the so-called [Me_2_pipdtH]I_3_ salt, differently from the previously cited adducts of R_2_dazdt, showed an exceptional reactivity toward platinum, producing the [Pt(Me_2_pipdt)_2_]I_6_ complex through a one-pot reaction in mild conditions.^22^ Further studies have been performed to better characterize the [R_2_pipdtH]I_3_ salts as well as to investigate the structure–property relationship of the systems based on R_2_pipdt and R_2_dazdt, in terms of both ligand interaction with halogens and leaching properties of the obtained products.

In this context, herein we report the results of the extensive studies performed over several years to provide support to the formation of the [Me_2_pipdtH]^+^ cation and the reason why Me_2_pipdt behaves so differently from R_2_dazdt toward diiodine. Specifically, the deep characterization of a series of I_3_^–^ salts, synthetically obtained by reacting R_2_pipdt (R = Me, **Me-1**; *^i^*Pr, *^**i**^***Pr-1**; Bz, **Bz-1**; Ph, **Ph-1**) ligands with diiodine in an organic solvent, was performed. The characterization at the solid state (mainly by single-crystal X-ray and neutron diffraction measurements) and in solution (electrospray ionization mass spectrometry (ESI-MS), UV–vis), well supported by density functional theory (DFT) calculations, was able to exclude the formation of a radical species while definitely pointing out the formation of the protonated [R_2_pipdtH]^+^ cations for **Me-1**, *^**i**^***Pr-1**, and **Bz-1** ligands. The finding of a different cation in the case of the **Ph-1** ligand, the **Ph-1C**^**+**^ cation resulting from an intramolecular condensation reaction, was also pointed out. A reasonable reaction mechanism for the formation of these salts, supported by DFT calculations, is also proposed and discussed to highlight why, in the case of the reaction of R_2_dazdt with diiodine, the suggested pathway does not occur.

## 2 Results and Discussion

As anticipated in Section 1 and highlighted in a previous paper of ours, X-ray diffraction measurements on the product, obtained by reaction between the Me_2_pipdt ligand (**Me-1**) with I_2_ in organic solvents, supported the formation of a triiodide salt, [Me_2_pipdt]I_3_ (**Me-1I**_**3**_), where the cationic moiety showed a highly symmetric [Me_2_pipdt]^+^ molecular structure.^22^ However, no evidence was found to support the radical nature of this cation, which should hold an odd number of electrons ([Me_2_pipdt]^•+^ = **Me-1**^**•+**^). This stimulated further studies to achieve a more reliable characterization of the salt. Moreover, open-shell DFT calculations ruled out the involvement of a radical cationic in **Me-1I**_**3**_ (see Section 2.1).

**Figure 1:**
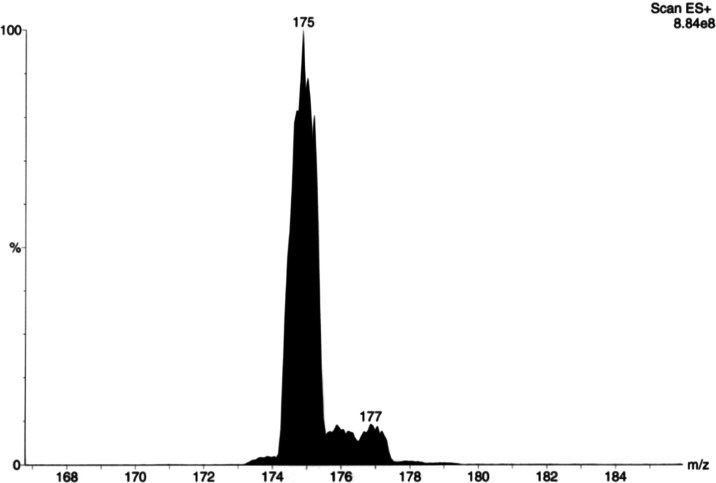
Electrospray-mass spectrum of the **Me-1I**_**3**_ salt in methanol solution, cationic part. See the Supporting Information for the anionic part of the spectrum (Figure S1) and the experimental details (Section S1).

The first evidence of a different cationic structure came from the ESI-MS measurements of the salt. Indeed, besides the detection of the triiodide anion (*m*/*z* 381 peak), the possible presence of a protonated [Me_2_pipdtH]^+^ (**Me-1H**^**+**^) species was supposedly based on the peak at *m*/*z* 175 (Figure 1).

^1^H NMR spectra of **Me-1** (Section S4 and Figure S7) exhibit two peaks at 3.75 and 3.57 ppm corresponding to the methylene and methyl protons, respectively. Both resonances are slightly downfield-shifted in the NMR spectrum of the salt **Me-1I**_**3**_, which in addition exhibits a broad band at 2.75 ppm. Since no broadening or large shifts of the peaks, as predictable for the presence of a radical ion, were observed, a possible involvement of a radical cationic species was ruled out. While the proton signal of the S–H group is missing, the broad band at the 2.75 ppm band may be indicative of a thiol–water exchange and supported by the known typical weakness of the S–H bond.^23,24^

Looking back at the original structure of the [Me_2_pipdt]I_3_ salt,^22^ we inferred that the existence of the **Me-1H**^**+**^ species does not disagree with the experimental structural data. As a matter of fact, the salt crystallizes in the symmetric **C**2/**c** space group and a *C*2 axis is passing just in the middle of the cation, making the two parts of the molecule identical. Considering the small scattering power of the hydrogen atom, the small asymmetry in **Me-1H**^**+**^ generated by the H^+^ could be easily overlooked in the diffraction experiment.

An alternative way of resolving this quandary was to decrease the symmetry in the cation, substituting the methyl groups with bulkier residues, namely, isopropyl (^*i*^Pr_2_pipdt, *^**i**^***Pr-1**), benzyl (Bz_2_pipdt, **Bz-1**), and phenyl (Ph_2_pipdt, **Ph-1**) groups. These ligands were prepared in agreement with ref (25) and, as previously made with **Me-1**, reacted with diiodine in a 1:2 molar ratio in CHCl_3_ at room temperature (see details in Section 4). As a result, by reacting the selected R_2_pipdt with diiodine in an organic solvent, the triiodide salt of the cationic ligand was invariably obtained at the solid state and fully characterized (see Section S3, Supporting Information).

**Figure 2:**
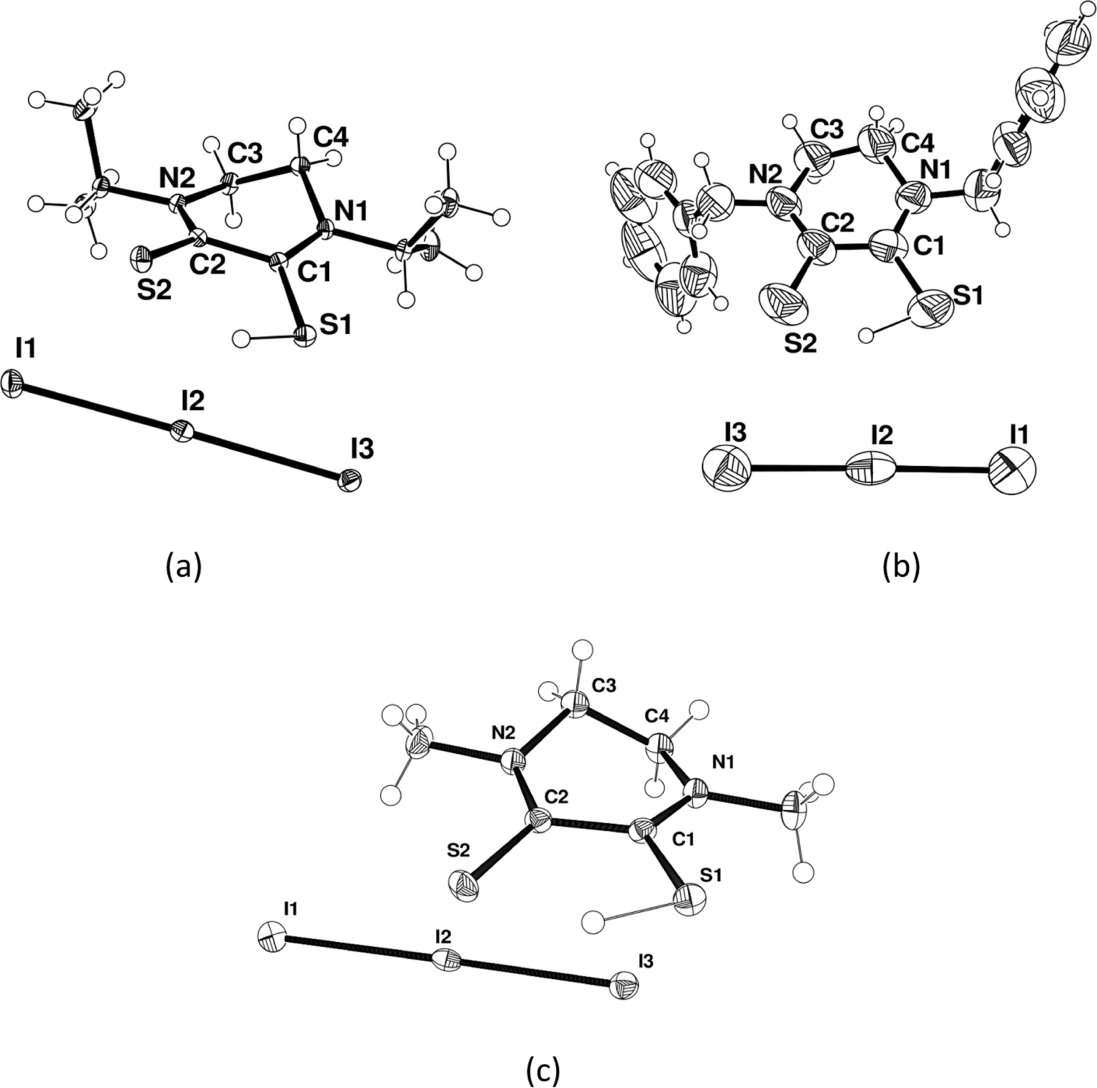
Molecular structure (ORTEP diagram) of *^**i**^***Pr-1I**_**3**_ (a), **Bz-1I**_**3**_ (b), and **Me-1I**_**3**_ (c) showing the atom labeling scheme. Thermal ellipsoids are shown at a 50% probability level. Additional data are provided in Section S5, Supporting Information.

Figure 2a,b shows that for *^**i**^***Pr-1** and **Bz-1** ligands, the corresponding I_3_^–^ salts crystallized in the *P*1̅ space group, hence without the *C*2 axis and with the asymmetric unit comprising the whole cation, differently from the previously cited **Me-1**. Indeed, in these two cases, a residual electron density was found away from one sulfur atom (S–H distances 1.22(3) and 1.60(6) Å and with C–S–H angles 90.5(15) and 87(2)°, respectively, for *^**i**^***Pr-1** and **Bz-1**). Thus, a hydrogen atom was reasonably associated with such an electron density. Selected bonds and angles are reported in Table 1.

**Table 1:** Selected Bond Lengths (Å) and Angles (deg)^a^

	**Me-1I_3_**	***^i^*Pr-1I_3_**	**Bz-1I_3_**	**Ph-1CI_3_**
C1–S1	1.709(2)	1.711 (2)	1.674(6)	1.700(4)
C10–S1				1.732(4)
C2–S2	1.672(2)	1.672 (2)	1.669(5)	1.648(4)
C1–C2	1.523(3)	1.527 (3)	1.519(8)	1.470(5)
C1–N1	1.308(3)	1.308 (2)	1.296(6)	1.311(5)
C2–N2	1.325(3)	1.328 (2)	1.306(7)	1.337(5)
N1–C1–C2	120.22(16)	119.80(16)	118.9(5)	124.5(3)
S1–C1–N1	120.47(15)	121.62(14)	123.2(5)	113.4(3)
C2–C1–S1	119.28(13)	118.57(13)	117.9(4)	122.0(3)
N2–C2–C1	116.57(16)	115.88(16)	118.4(5)	114.4(3)
C1–S1–C10				90.2(2)
S2–C2–N2	124.37(15)	125.11(15)	123.1(5)	127.3(3)
C1–C2–S2	119.06(14)	118.95(13)	118.6(4)	118.2(3)
C1–S1–H1	88.0(12)	90.5(15)	87(2)	
I1–I2–I3	178.025(6)	177.915(6)	178.925(19)	176.990(14)

a Additional data are provided in Section S5, Supporting Information.

A further attempt to ascertain the presence of the thiolic hydrogen atom in the crystal structure of **Me-1I**_**3**_ was made by collecting single-crystal X-ray diffraction data at low temperature (100 K) to improve the intensity and resolution of the diffraction data and reducing the thermal motion, thus allowing the identification of subtle features in the electron density. The redetermination of the crystal structure of **Me-1I**_**3**_ led to a change in the space group symmetry from that previously reported **C**2/**c** to the new *P*2_1_/*c*. The discrepancy between the two symmetry group assignments was attributed to overlooked weak reflections rather than to a disorder–order transition. The new model showed no diffraction peak violations and was refined with the overall atom connectivity and molecular symmetry in agreement with the presence of ordered hydrogen on one sulfur atom. All of the hydrogen atoms, including the proton of the S–H group, were localized in the difference Fourier map calculated from X-ray data at low temperature, as shown in Figure 2c.

The nature of the bonding in the dithio-piperazinium cation **Me-1H**^**+**^ can be discussed in detail based on its structural features in terms of bond lengths and bond angles, as described below. A similar analysis can be performed for the other presented structures that exhibit similar molecular and packing features.

The planarity of two thioamide moieties is indicative of an sp^2^ character of piperazine N atoms and consequently of at least partially double bond nature of the C–N amidic bonds. However, the C–S (1.709(2) Å) and C–N (1.308(3) Å) bond lengths of the thio-amidium units (−N=C–SH) are very close to the values typical for a C(sp^2^)–S single bond and a C(sp^2^)=N(sp^2^) double bond, respectively; thus, the configuration appears well defined. Instead, the C–S and C–N bond distances of the thion-amide groups (1.672(2) and 1.325(3) Å) are very close to the values observed in the free ligands (1.668(2) and 1.325(2) Å)^26^ as well as in other analogous noncyclic ligands and in many transition-metal dithiooxamide complexes where the ligand acts as a neutral bidentate donor group.^27,28^ Therefore, the two N–C–S groups can be better described as an admixture of thiol and thione resonance structures. Moreover, the C(1)–C(2) bond distance (1.523(3) Å) between the two thioamide moieties is at the upper limit of the range of values accepted for a C sp^2^–C sp^2^ single bond, while the C(3)–C(4) bond length (1.504(3) Å) is slightly shorter than the expected value for a C sp_3_–C sp^3^ connection. Finally, C(3)–N(2) and C(4)–N(1) distances (1.470(3) and 1.474(3) Å) are in good agreement with the expected values for such bonds.

Another important aspect regards the conformation adopted by the piperazine ring that is strictly connected with the dihedral angle θ between the planes comprising the thioamide moieties (N–C–S) and with the torsional angles (N–C–C–N) along the ring. The substantially smaller dihedral angles θ and the torsion angles around the central C–C bonds in the dithio-piperazinium cation (9.80, 10.89°) compared to those observed in the corresponding neutral ligand (35.28 and 34.36°) could be reasonably due to the presence of orienting intramolecular and intermolecular interactions involving the thiolic hydrogen and the triiodide ion.

**Figure 3:**
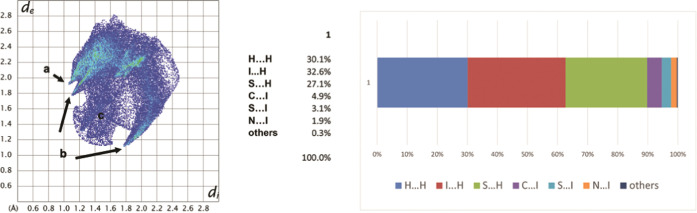
Fingerprint plot (left) and percentage distribution of different interactions (right) present in **Me-1I**_**3**_ as obtained from fingerprint plots.

The main intermolecular interactions in the crystal structure of **Me-1I**_**3**_ were analyzed using the Hirshfeld surface mapped with *d*_norm_(28) and the corresponding two-dimensional fingerprint plots (Figure 3).^29^ The analysis shows that the dominant interactions in the packing are H···H, H···I, and H···S. The H···I interactions are represented by the upper short spike (labeled **a** in Figure 3), in the bottom left area of the fingerprint plot. This spike is not only due to the short contact of the central iodine of I_3_^–^ with the thiolic hydrogen atom (2.98(3) Å) but also due to multiple interactions between the lateral atoms of triiodide with the C–H groups of the ring and those of the methyl substituent on the amidic nitrogen. The lower pair of short spikes labeled **b** in Figure 3 represents the H···S intermolecular interactions. The spikes are due to several contacts of different lengths, involving both sulfur atoms, that overlap in the pattern.

In the fingerprint plots, many points reaching down to (1.2, 1.2) along the plot diagonal arise from the shortest H···H contacts (labeled **c** in Figure 3). These contacts give a strange broad diffuse region of blue points ranging from 1.2 to 1.6 Å due to multiple contacts between the C–H hydrogen atoms of adjacent molecules.

Although the positions of the thiolic protons were reliable enough to safely rule out other possible models, we decided to perform a single-crystal neutron diffraction study to obtain a more accurate determination of the hydrogen sites.

However, also in this case, the data were not good enough to allow a complete refinement of the structural parameters, as described in Section 4. Nevertheless, the crystal data obtained by these last measurements, **Me-1I_3_*n***, were good enough to lead to a reliable position of the hydrogen and refine the crucial data: the thione moiety is confirmed to be protonated at the sulfur atom with the S–H bond distance (1.34(3) Å) in good agreement with the other values from neutron data reported in the literature.^30^

**Figure 4:**
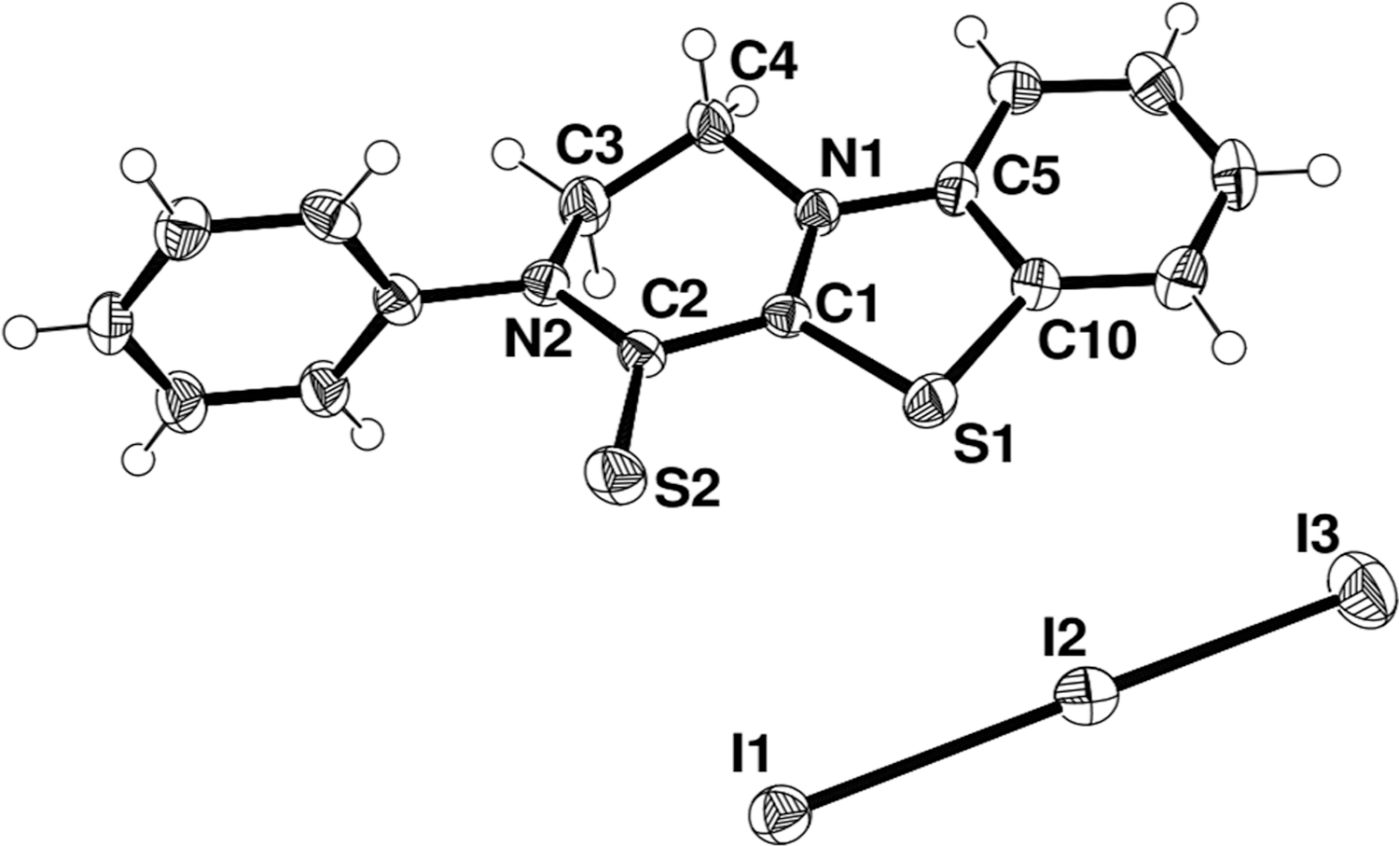
Molecular structure (ORTEP diagram) of [Ph_2_pipdt-C]I_3_ (C = condensed; **Ph-1CI**_**3**_) showing the atom labeling scheme (corresponding selected bond lengths and angles are reported in Table 1). Thermal ellipsoids are shown at a 50% probability level. Additional data are provided in Section S5, Supporting Information.

A further contribution to understanding these systems came from the results of the reaction between the ligand **Ph-1** and diiodine under the same conditions reported for the other compounds. Indeed, instead of the expected [Ph_2_pipdtH]^+^, a different structure for the cation, namely, **Ph-1C**^**+**^, possibly deriving from an intramolecular condensation reaction, was found, as shown in Figure 4 and detailed in Section 4.1.

As shown by the molecular structure, one sulfur atom is bonded with the ortho carbon atom of the neighboring phenyl group forming a new five-membered S,N-heterocycle in a benzothiazolylium cationic moiety. A radical mechanism mediated by iodine can be reasonably invoked for the reactions of **R-1** with I_2_, as confirmed by experiments with the radical trapping (2,2,6,6-tetramethylpiperidin-1-yl)oxyl (TEMPO) (see Section S7, Supporting Information).

These results stimulated further studies on the formation mechanism of the **R-1(H)I**_**3**_ salts and a comparison with the corresponding reaction undergone by **R-2** ligands.

### 2.1 DFT Calculations and Reaction Mechanism Hypothesis

**Figure 5:**
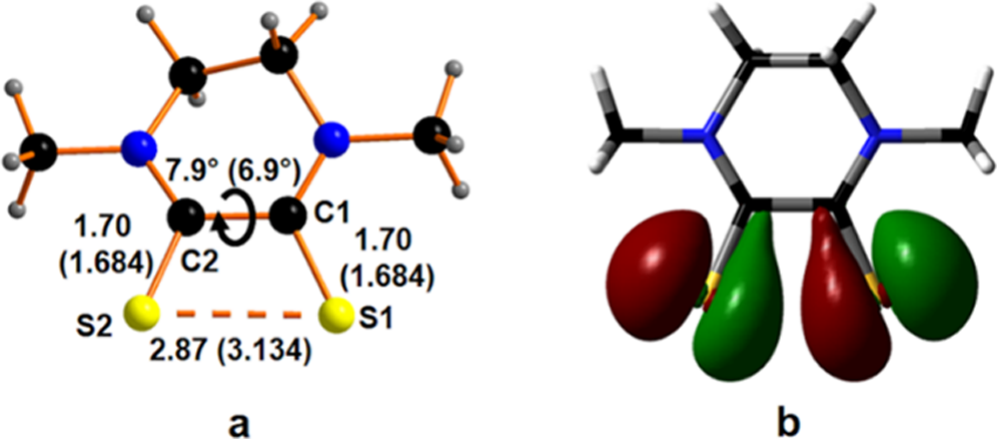
(a) Calculated structure of the **Me-1**^**•+**^ cation. Calculated *vs* measured from X-ray structure (Refcode QIPYIX^22^) bond distances. (b) Calculated singly occupied molecular orbital (SOMO).

Results from open-shell DFT calculations have contributed to ruling out the possible involvement of a radical cationic species **Me-1**^**•+**^ in **Me-1I**_**3**_. The computational analysis on a potential radical **Me-1**^**•+**^ species did not accurately reproduce the available X-ray experimental structure since, despite a good agreement for the S1–C1–C2–S2 dihedral angle, the calculated S1–S2 distance seems to be particularly shorter (2.87 Å) compared to the available experimental one (3.134(4) Å) in the original reported structure (Refcode QIPYIX), as shown in Figure 5.^22^ The calculated S–S distance was indeed found in the range of a half-bond as it was highlighted by some of us in previous works^31−33^ and close to the value of 2.8168(11) reported for the half-bond S–S distance in the radical cation of 1,8-chalcogen naphthalenes.^34^ On the other side, the experimental S–S distance larger than 3 Å can be considered nonbonding.

The occurrence of the half-bond in the calculated structure of **Me-1**^**•+**^ can be explained by looking at the highest occupied molecular orbital (HOMO) (shown in Section S2, Supporting Information) of **Me-1** characterized by an S1···S2 antibonding interaction. The removal of one electron from the HOMO, which generates the SOMO of **Me-1^•+^** (Figure 5b), weakens the antibonding character, allowing the formation of the S–S half-bond (see Figure S2 for calculated frontier orbitals). Such a behavior is also confirmed by the optimization of the **Me-1**^**2+**^ species, obtained upon the removal of a second electron, generating the **Me-1**^**2+**^ species, where a single S–S bond is found (calculated distance 2.22 Å; see Figure S3). On the other side, a very close agreement between the calculated (3.24 Å) and experimental (3.134 Å) S1···S2 distances, as well as between the S1–C1–C2–S2 dihedral angles, was obtained for the closed-shell [Me_2_pipdtH]^+^ species, **Me-1H**^**+**^, confirming the ESI-MS observation for the presence of the protonated species in the solid state. We also excluded the protonation of one of the nitrogen atoms, the N-protonated isomer being 21.9 kcal mol^–1^ (91.8 kJ mol^–1^) higher in energy compared to the S-protonated one (see Figure S4).

**Figure 6:**
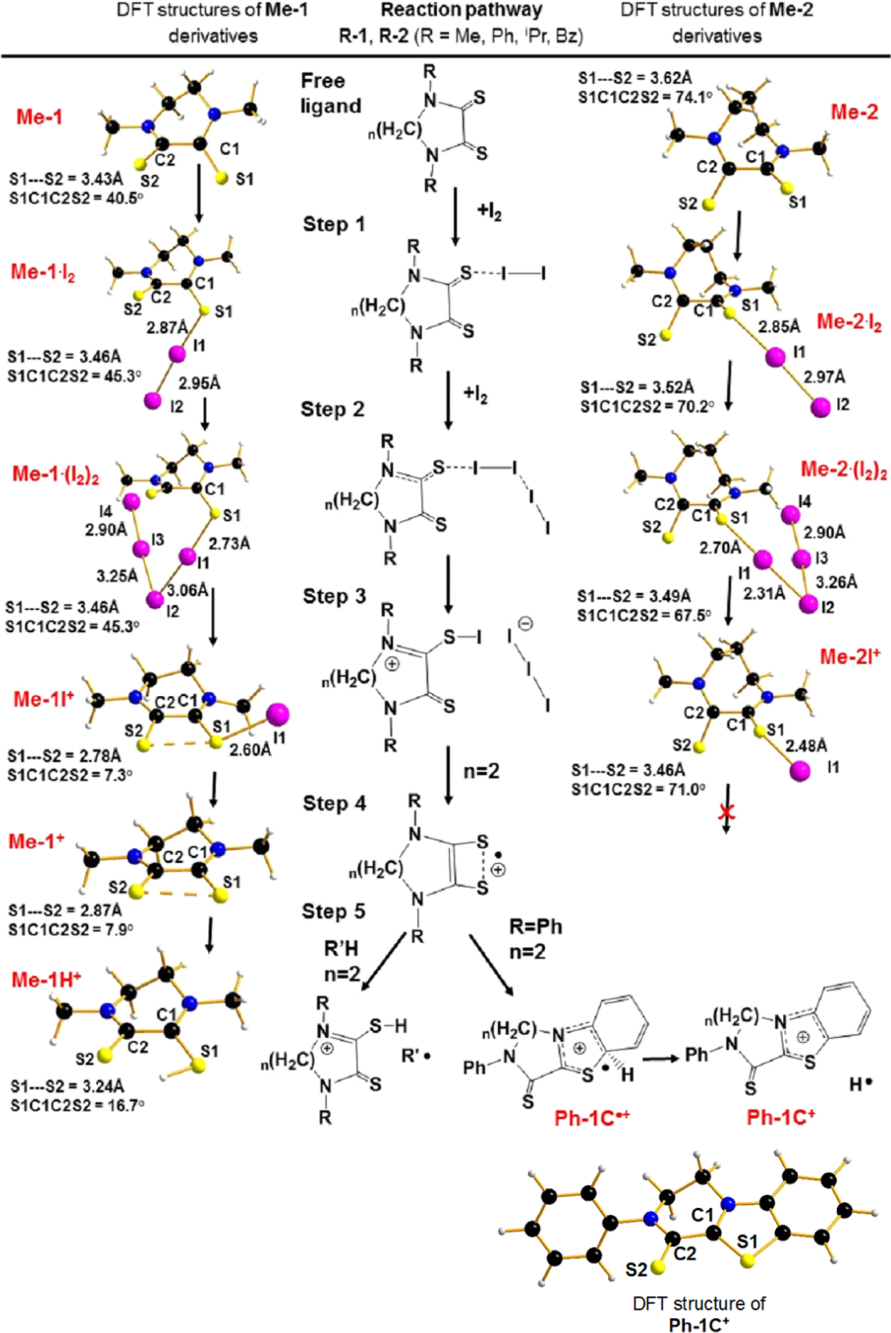
Proposed reaction mechanism for the formation of **Me-1H**^**+**^ and **Ph-1C**^**+**^ species and comparison with the corresponding **Me-2** system based on the calculated structures in the CHCl_3_ solvent.

DFT calculations have also been employed to correlate the observed different reactivity of **R-1** and **R-2** ligands, only differing by a −CH_2_– in the cycle, with their different structural conformation, and to elaborate a reasonable reaction mechanism. Accordingly, the reaction pathway for the general **R-n** with *n* = 1 or 2 in CHCl_3_ solvent was obtained (Figure 6). In the present discussion, only the structural data from the optimized structures are used.

In outlining a possible reaction pathway for the transformation of **R-n** ligands in the corresponding triiodide salts, it seems reasonable to invoke −S···I_2_ halogen-bonded species of D···I_2_ type. As anticipated in Section 1, depending on the donor–diiodine interaction strength, the electronic distribution changes compared to that in the free reagents, and a lengthening of the I–I distance proportional to the shortening of the intermolecular distance was observed.^11^ In the presence of an excess of I_2_, another free I_2_ molecule may interact with the other S atom or, as demonstrated by previous studies, may promote the heterolytic I–I cleavage of the already involved I_2_ molecule. The net result is the formation of halogen-bonded species SI^+^···I_3_^–^ with the SI^+^ acting as halogen-bonding donor and the corresponding triiodide salt.^35−38^

The optimized structures in the CHCl_3_ solution pointed out that the energy of the two possible isomers, alternatively involving two or one sulfur center(s), differs by less than 1 kcal mol^–1^ (4 kJ mol^–1^) in both cases. Based on these results, it is reasonable that both isomers are present in the solution. For our purposes, we focused on the role of the second I_2_ molecule as the cleavaging species for the I–I bond. In that case, the second I_2_ molecule interacts through a halogen bond with the terminal I-atoms of the chain. The following step is the heterolytic cleavage of the I1–I2 bond, resulting in an I_3_^–^ anion and a ligand–I^+^ cation, as well demonstrated in the case of 1,3-bis(2,6-diisopropylphenyl)-1,3-dihydro-2*H*-imidazole-2-thione in the presence of one and two iodine molecules.^39^

Taking the above as typical behavior of soft donor–acceptor systems, it has been applied to both the cited classes of ligands, specifically indicated as **Me-1** and **Me-2**.

**Figure 7:**
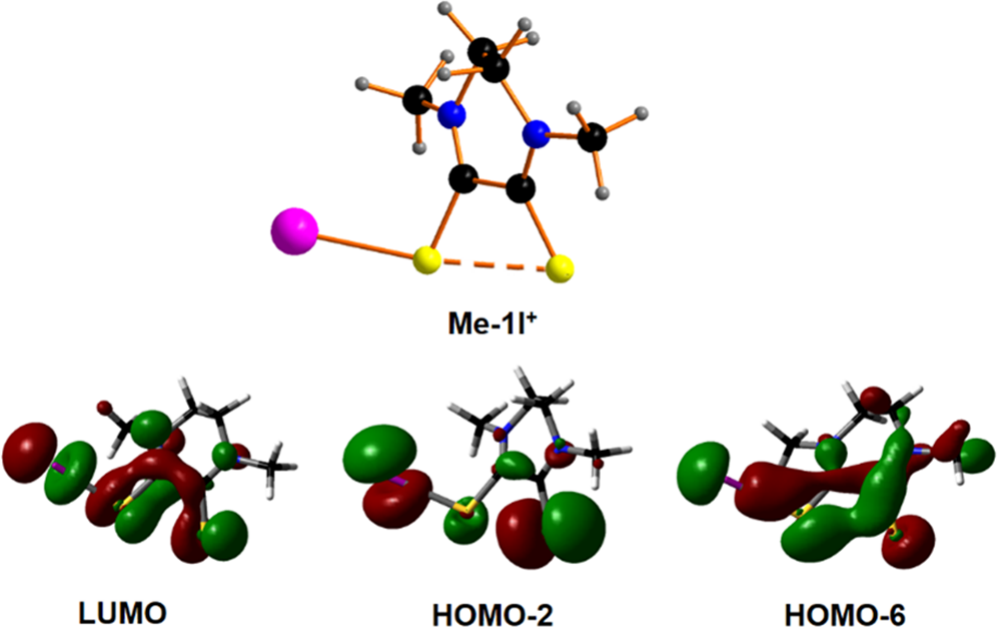
Three LUMO, HOMO–2, and HOMO–6 orbitals of **Me-1I**^**+**^ supporting the three-center-four-electron hypervalent bonding scheme for the S–S–I group.

As shown in Figure 6, starting from the free ligand, the reaction pathway consists of five main steps. It is worth noting by comparing the two ligands that the main structural difference for **Me**-**1** and **Me-2** is in the value of the dihedral S–C–C–S angle calculated as 40 and 74°, respectively. In the first step, the formation of an L–I_2_ halogen-bonded adduct is considered. The main geometrical difference between **Me**-**1·I**_**2**_ and **Me-2·I**_**2**_ remains in the S–C–C–S angle (45 *vs* 70° for **Me-1·I**_**2**_ and **Me-2·I**_**2**_, respectively). Furthermore, the I1–I2 bonds are partially elongated compared to the free I_2_ molecule (*e.g.*, 2.95 Å **Me-1·I**_**2**_ against 2.74 Å), while the S1–I1 distance is around 2.87 and 2.85 Å for **Me-1·I**_**2**_ and **Me-2·I**_**2**_, respectively. The next step is the interaction of a second I_2_ unit with the coordinated one, which causes a weakening of the I1–I2 bond and the strengthening of the S1–I1 bond. As a consequence, in **Me**-**1·(I**_**2**_**)**_**2**_ and **Me**-**2·(I**_**2**_**)**_**2**_ adducts, the I1–I2 distance elongates up to 3.11 Å and a linear I_3_^–^ group seems to be almost ready to leave the compound. The S1–I1 distances are found to be 2.73 and 2.70 Å for **Me**-**1·(I**_**2**_**)**_**2**_ and **Me**-**2·(I**_**2**_**)**_**2**_, respectively. Step 3 reports the calculated structures of the **Me**-**1I**^**+**^ and **Me-2I**^**+**^, where the I_3_^–^ group leaves the molecular compound. For **Me-2I**^**+**^, the S1–I1 distance is 2.48 Å and the C1–S1–I1 angle is 107° (with an S–C–C–S dihedral angle of 71°). Calculated values for I1–I2 distances in **Me-1·(I**_**2**_**)**_**2**_ and **Me-2·(I**_**2**_**)**_**2**_ and S1–I1 in **Me-2I**^**+**^ well agree with literature crystal data for other similar species (see the corresponding specie obtained by 1,3-bis(2,6-diisopropylphenyl)-1,3-dihydro-2*H*-imidazole-2-thione, as a reference).^39^ On the contrary, **Me-1I**^**+**^ presents several unusual features. First, the S1–I1 distance is 2.60 Å, 0.12 Å longer than in **Me-2I**^**+**^ and in a typical S–I single bond. More interestingly, the S1–S2 distance is as short as 2.78 Å, a distance typical of a half S–S bond. For comparison, the S1–S2 distance in **Me-2I**^**+**^ is longer than 3.40 Å. Moreover, the S1–C1–C2–S2 dihedral angle is almost flat (7.3°) and the S2–S1–I1 angle is 155°, close to the linearity. The geometrical arrangement in **Me-1I**^**+**^ is in agreement with a three-center-four-electron hypervalent system description.^40−42^ Specifically, the HOMO–6, HOMO–2, and lowest unoccupied molecular orbital (LUMO) combinations are involved in the S–S–I bond, almost mimicking a classical Rundle–Pimentel model by the occurrence of one bonding, one nonbonding, and one antibonding molecular orbitals, respectively, as shown in Figure 7. The nonlinear arrangement of the S–S–I group affects the shape of the nonbonding HOMO–2 orbital to the classical symmetric case of I_3_^–^, showing on the I atom a relevant contribution of a perpendicular orbital by the bond axis. It is noteworthy, in the case of **Me-2I**^**+**^, that no hypervalent S–S–I bond occurs, the dihedral S–C–C–S angle being too wide.

**Figure 8:**
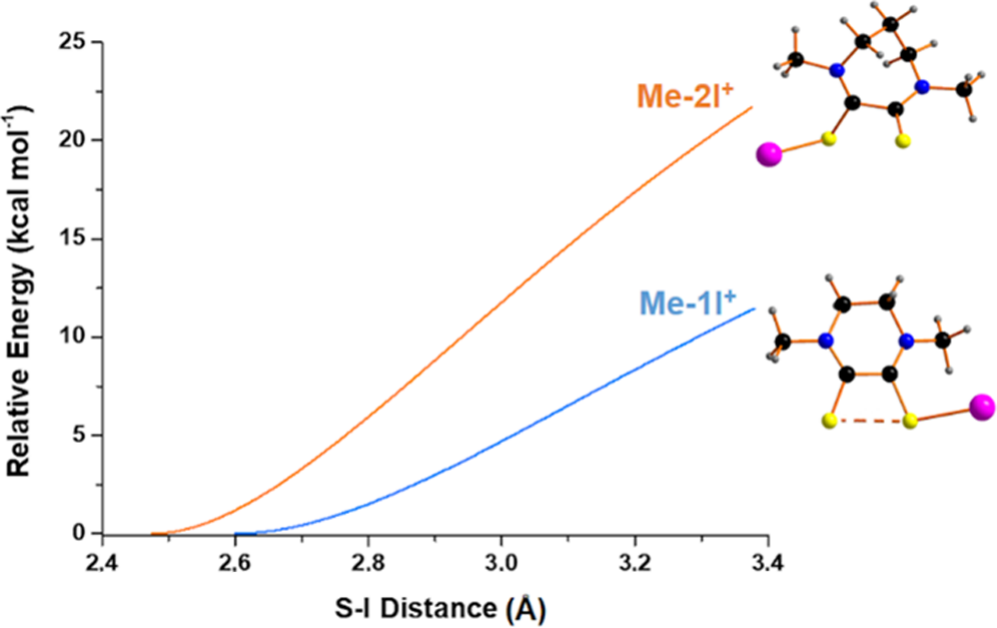
Plots of the energy for the elongation of the S–I bonds for **Me-1I**^**+**^ and **Me-2I**^**+**^ relative to the equilibrium distances in the CHCl_3_ solvent.

Accordingly, in the case of **Me-1**, the presence of the hypervalent bond suggests the irreversible homolytic splitting of **Me-1I**^**+**^ in two radicals, namely, **Me-1**^**•+**^ and **I**^**•**^. Remarkably, as highlighted in Figure 8, the energy required for this splitting is less than half for **Me-1I**^**+**^ with respect to **Me-2I**^**+**^.

**Figure 9:**
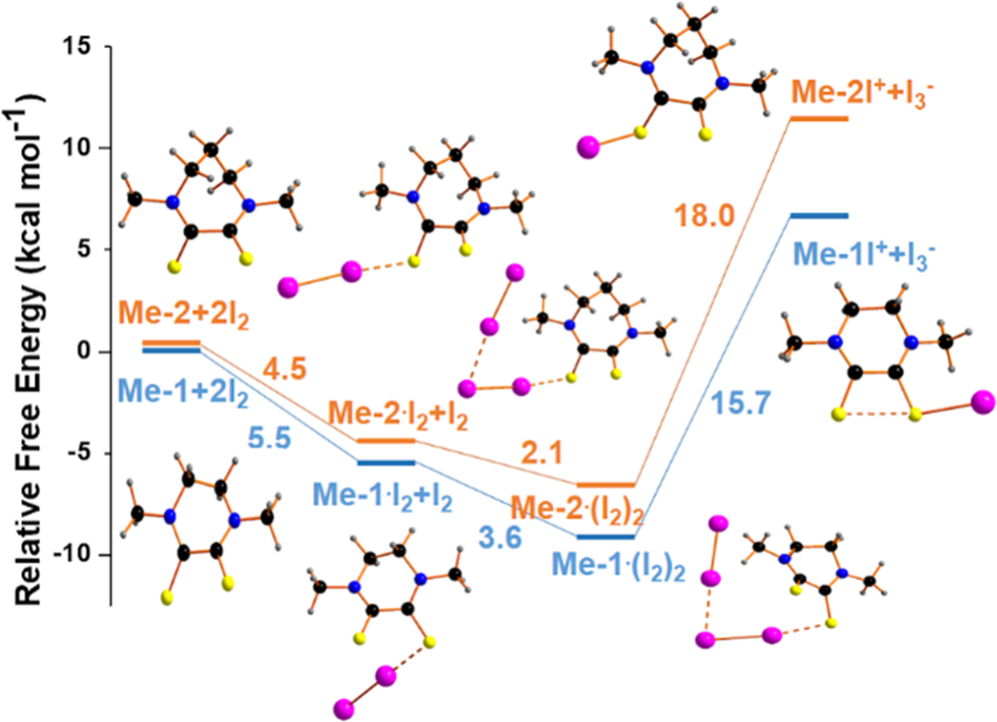
Comparison of the free-energy profile for the interaction with iodine of **Me-1** and **Me-2** ligands in the CHCl_3_ solvent. The reference energy has been calculated concerning the noninteracting molecules of **Me-*N*** ligands (*N* = 1 and 2) and iodine. Free iodine molecules and I_3_^–^ ions are omitted in the picture for clarity. A 15.6 kcal/mol (65.3 kJ mol^–1^) endergonic homolytic scission of **Me-1I**^**+**^ in **Me-1**^**•+**^ and **1**^**•**–^ has been found by calculations.

The calculated free-energy profiles, shown in Figure 9, point out the significantly more favored formation of **Me-1I**^**+**^ than the corresponding **Me-2I**^**+**^ species, attributable to the presence of the hypervalent bond in **Me-1I**^**+**^.

In the final step, the recombination of the radical species is considered. Specifically, **Me-1**^**•+**^ will compete with an R–H for an H^**•**^ extraction to provide the **Me-1H**^**+**^ structure. The most plausible R–H source of the radical H^**•**^ is the CHCl_3_ solvent. The same behavior is expected for similar systems based on *^i^*Pr- and Bz-substituents. In the case of the phenyl-substituted **Ph-1**, **Ph-1**^**•+**^ will go for an internal condensation reaction to produce the radical intermediate **Ph-1CH**^**•+**^. The latter, featuring a tetrahedral carbon center, may interact with radical species in solution to provide the final species **Ph-1C**^**+**^ together with some radical recombination products. It is worth noting that the **Ph-1CI**_**3**_ condensation product is an indirect proof of the existence of an **R-1**^**•+**^ specie in solution where the phenyl group is acting as an internal trapping radical. Besides, I^**•**^ may combine with R^**•**^ and/or with I^–^ or I_3_^–^ to form I_2_^**•**–^, followed by a disproportion reaction to I_3_^–^ and I^–^, in agreement with the previous finding by other authors.^43,44^

To summarize, despite the possibility for the two systems to evolve toward the **R-*N*·2I**_**2**_ (*N* = 1, 2) adducts, in the case of **R-1** ligands, the irreversible formation of an **R-1**^**•+**^ radical is energetically feasible shifting the reaction toward the formation of the triiodide salts. On the contrary, for **Me-2** only an equilibrium between the free ligand and various iodine adducts is envisaged due to the energetically disfavored formation of the radical species.

As a consequence of the above, we can state that the difference in the torsion angle of the free ligands has been proved to represent the structural key factor in determining the reactivity of the two systems.

## 3 Conclusions

The reaction of the hexa-atomic cyclic dithiooxamide ligands (R_2_pipdt, **R-1**) with diiodine, unexpectedly providing triiodide salts, has been thoroughly revisited and newly investigated. Extensive experimental work and new theoretical interpretative frameworks were required for shedding light on the structural features of the reaction products and the reaction mechanism behind the triiodide salt formation. At the end of this journey, X-ray diffraction measurements, complemented by neutron diffraction experiments, were able to experimentally establish the main formation of S-protonated ligands upon the reaction of **R-1** ligands (with R = alkyl and Bz) with iodine in organic solvents. Differently, a new five-membered S,N-heterocycle in a benzothiazolylium cationic moiety was formed in the case of R = Ph. DFT calculations, based on the structural data of **Me-1** and **Me-2**, allowed us to speculate on a reasonable reaction mechanism. Specifically, when halogen bonding occurs with I_2_ in the case of **Me-1**, the almost planarity of the S–C–C–S moiety allows the formation of a three-center-four-electron S–S–I bond, which irreversibly evolves toward active radical species through the homolytic cleavage of the S–I bond. Eventually, the radical ligand **R-1**^**•**^ induces an intra- (as for R = Ph) or inter- (as for the other R substituents) molecular radical reaction with the formation of the corresponding I_3_^–^ salts. Otherwise, in the case of the **Me-2**, the wider S–C–C–S torsion angle does not allow the formation of the three-center-four-electron S–S–I bond hindering the radical activation with the consequence that only **Me-2·nI**_**2**_ halogen-bonded adducts occur.

Thus, the reported combined experimental and computational study allowed us to elaborate on a reasonable reaction mechanism providing an interesting example of a halogen-bonding radical reaction promoted by diiodine. This opens the way to further investigations on the peculiar reactivity demonstrated by **R-1HI**_**3**_ salts toward metals, primarily platinum.

## 4 Experimental Section

### 4.1 Materials and Methods

Reagents and solvents of reagent-grade quality were used as supplied by Sigma-Aldrich. The R_2_pipdt ligands **R-1** were prepared according to ref (25). Ph_2_pipdt was synthesized as described in the following section.

### 4.2 Synthesis of Ph_2_pipdt and R_2_pipdt/Triiodide Salts

#### 4.2.1 Ph-1

*N*,*N*′-Diphenyl-ethylenediamine (Ph-NH-(CH_2_)_2_-NH-Ph, 10 g, 0.048 mol) dissolved in dichloromethane (CH_2_Cl_2_, 100 mL) and oxalyl chloride ((COCl)_2_, 7.2 g, 0.056 mol) in CH_2_Cl_2_ (100 mL) was contemporarily dropped into 600 mL of CH_2_Cl_2_ containing triethylamine (Et_3_N, 4 mol). The product was washed with water three times and then anhydrified and purified with activated charcoal. The solution was hence dried under vacuum providing a white viscous product that was washed several times with acetone. The residue was recrystallized by CH_2_Cl_2_/petroleum ether (60–80°), obtaining the expected cyclic diketone (2.57 g, 0.0096 mol, yield: 20.1%).

MIR (on KBr pellets, cm^–1^): 3343vw, 3045w, 3024w, 3003vw, 2941w, 1679vs, 1593m, 1492s, 1472m, 1456w, 1424s, 1397s, 1358s, 1328w, 1298s, 1244m, 1208w, 1182w, 1144m, 1084m, 1070m, 983m, 903vw, 799w, 760s, 692s, 644w, 554w, 498m, 422w.

The intermediate was reacted for 15 min with Lawesson’s reagent in a 1:1.2 molar ratio (4.7 g, 0.012 mol) in refluxing toluene under stirring. After the reaction, the solution was dried, and then the brown residue was washed several times with MeOH and recrystallized by dissolution in hot tetrahydrofuran (THF) and slowly cooling down the solution. Well-shaped brown-red crystals of the **Ph-1** ligand, suitable for single-crystal X-ray diffractometry, were obtained in yields spanning from 33 to 45%.

C% 64.90; H% 4.83; N% 9,75; S% 21.13; calcd for C_16_H_14_N_2_S_2_ (FW: 298.433 g mol^–1^): C% 64.39; H% 4.73; N% 9.39; S% 21.49. MIR (on KBr pellets, cm^–1^): 3045vw, 3024vw, 3003vw, 2941vw, 2911 vw, 1653w, 1590s, 1489vs, 1435vs, 1346vs, 1320s, 1276s, 1203mw, 1160m, 1100w, 1071mw, 1041m, 1000w, 900m, 782mw, 764s, 692s, 635w, 623m, 610w, 582w, 551m, 485w. Raman (cm^–1^): 3059vs, 3037s, 2986s, 2966m, 2924mw, 2898m, 1589s, 1416m, 1345mw, 1267mw, 1227s, 1161s, 1072m, 1001vs, 958w622mw, 554mw, 485m, 298w, 274m, 236w, 205w, 148m, 132s-sh, 106vs, 84m-sh. 74m. UV–vis (in CH_2_Cl_2_) [λ = nm (ε = dm^3^ mol^–1^ cm^–1^)]: 274-sh, 333(11 000), 439(800). ^1^H NMR (CDCl_3_, 500 MHz) δ (ppm): 7.51 (m, 4H), 7.40 (m, 2H), 7.38 (m, 4H), 4.18 (s, 4H, CH_2_).

#### 4.2.2 R-1HI_3_

In a typical reaction, 0.300 g of R_2_pipdt (R = Me, *^i^*Pr, Bz) was dissolved in 100 mL of CHCl_3_ and reacted with I_2_ in a 1:2 molar ratio (appropriate amount of I_2_ in 250 mL of CHCl_3_). Well-shaped crystals of **R-1HI_3_**, suitable for X-ray studies, were obtained from the solution by slow solvent evaporation and washed with petroleum ether (40–60°).

#### 4.2.3 Me-1HI_3_

An almost quantitative yield of needle-shaped dark brown crystals was obtained. Anal. found: C% 13.19; H% 1.93; N% 5.06; S% 11.55; calcd for C_6_H_11_N_2_S_2_I_3_ (FW: 555.928 g mol^–1^): C% 12.96; H% 1.99; N% 5.04; S% 11.54. ESI-MS *m*/*z* [M + H]^+^ calcd: 175.04, found: 175 (100%); [I_3_]^−^ calcd and found: 380.71; see further details in Section S1, Supporting Information. MIR (on KBr pellets, cm^–1^): 2965mw, 2925m, 2860w, 1541s-br, 1431vw, 1404vs, 1383vs, 1365vs, 1260vw, 1248mw-br, 1207mw, 1105s-br, 1045vw, 1015s, 900vw, 665vw, 535w, 456vw, 420vw, 394w, 385vw, 368w, 349m, 340w, 322m, 313mw, 300m, 274s. Raman (cm^–1^): 2972mw, 2909m, 2843w, 2183vw, 1900vw-br, 1562w, 1441mw, 1366mw, 1284w, 1248mw, 1142mw, 1106m, 672mw, 538m, 426w, 324w, 224m, 112vs. UV–vis (in CH_3_CN) [λ = nm (ε = dm^3^ mol^–1^ cm^–1^)]: 292(50 000), 362(24 000). ^1^H NMR (CD_3_CN, 500 MHz) δ (ppm): 3.78 (s, 4H), 3.53 (s, 6H); see further details in Section S4, Supporting Information.

#### 4.2.4 *^i^*Pr-1HI_3_

An almost quantitative yield was obtained in product collection. Anal. found: C% 20.36; H% 3.09; N% 4.68; S% 10.47; calcd for C_10_H_19_N_2_S_2_I_3_ (FW: 612.108 g mol^–1^): C% 19.62; H% 3.13; N% 4.58; S% 10.48. ESI-MS *m*/*z* [M + H]^+^ calcd: 231.10, found: 231.26 (100%); [M_2_]Na^+^ calcd: 483.78, found: 483.36 (18%); [M_2_ – S]Na^+^ calcd: 451.71, found: 451.30 (27%); [M_2_ – 2S]Na^+^ calcd: 419.65, found: 419.39 (50%); [I_3_]^−^ calcd and found: 380.71. MIR (on KBr pellets, cm^–1^): 2971m, 2927w, 2866vw, 1557m, 1505s, 1454m, 1364vs, 1280vw, 1247w, 1208vw, 1180s, 1123w, 1098s, 923vw, 882w, 791vw, 683vw, 589m, 458vw. Raman: the sample showed fluorescence. However, the most intense peaks at 2973, 2929, and 116 cm^–1^ can be observed. UV–vis (in CH_3_CN) [λ = nm (ε = dm^3^ mol^–1^ cm^–1^)]: 296(40 000), 364(21 000). ^1^H NMR (CD_3_CN, 400 MHz,) δ (ppm): 5.26 (hept, *J* = 6.7 Hz, 1H), 3.59 (s, 2H), 1.31 (d, *J* = 6.7 Hz, 6H).

#### 4.2.5 Bz-1HI_3_

An almost quantitative yield was obtained in product collection. Anal. found: C% 30.69; H% 2.68; N% 4.01; S% 9.24; calcd for C_18_H_19_N_2_S_2_I_3_ (FW: 708.196 g mol^–1^): C% 30.53; H% 2.70; N% 3.96; S% 9.06. ESI-MS *m*/*z* [M + H]^+^ calcd: 327.10, found: 327.15 (37%); [M_2_–S]^+^ calcd: 621.21, found: 621.27 (21%); [M_2_–S]^2+^ calcd: 311.11, found: 311.19 (100%); [M_2_–S]Na^+^ calcd: 643.19, found: 643.28 (40%); [I_3_]^−^ calcd and found: 380.71. MIR (on KBr pellets, cm^–1^): 3401m-br, 2972s, 2926m, 2859w, 1672m-sh, 1647s, 1525s-sh, 1493s, 1473s-sh, 1456s-sh1426m, 1365vs, 1285mw, 1259mw, 1224w, 1183vs, 1122m, 1099m, 906w, 883w, 762mw, 731mw, 682w, 654w, 590mw, 532w. ^1^H NMR (CD_3_CN, 400 MHz) δ (ppm): 7.45–7.27 (m, 10H), 5.29 (s, 2H), 4.65 (s, 2H), 3.65–3.54 (m, 2H), 3.51–3.40 (m, 4H).

#### 4.2.6 Ph-1CI_3_

An almost quantitative yield was obtained in the product following the same reaction conditions described above. Anal. found: C% 28.65; H% 1.27; N% 4.20; S% 9.74; calcd for C_16_H_13_N_2_S_2_I_3_ (FW: 678.126 g mol^–1^): C% 28.33; H% 1.93; N% 4.13; S% 9.45. ESI-MS *m*/*z* [M + H]^+^ calcd: 297.05, found: 297.16 (100%); [I_3_]^−^ calcd and found: 380.71. MIR (on KBr pellets, cm^–1^): 3045w, 3024vw, 3003vw, 2941w, 2911 vw,1577m, 1505w, 1490s, 1469vs, 1450m, 1354m, 1331s, 1288ms, 1251ms, 1223m, 1110vw, 1070w, 1030vw, 760m, 749vs, 710w, 691s, 626w, 590w, 553w, 458vw. Raman: 3055m, 2097w, 1510m, 1439vw, 1401w, 1380vw, 1359m, 1287w, 1251w, 1100w, 1073w, 1003w, 553w, 155s, 113 *vs* UV–vis (in CHCl_3_) [λ = nm (ε = dm^3^ mol^–1^ cm^–1^)]: 257(6000), 311(12 000). ^1^H NMR (CD_3_CN, 500 MHz) δ (ppm): 8.37 (dt, *J* = 8.3, 0.9 Hz, 1H), 8.24 (dt, *J* = 8.5, 0.8 Hz, 1H), 8.06 (ddd, *J* = 8.5, 7.3, 1.2 Hz, 1H), 7.96 (ddd, *J* = 8.3, 7.3, 1.0 Hz, 1H), 7.64 (m, 2H), 7.56 (m, 3H), 5.06 (m, 2H), 4.55 (m, 2H). ^13^C NMR (151 MHz, CD_3_CN) δ: 176.75, 167.26, 144.21, 142.15, 134.95, 132.00, 130.92, 130.70, 130.05, 126.24, 124.94, 118.65, 51.31, 46.02.

### 4.3 Chemical and Spectroscopical Characterization

Microanalysis was performed on a Carlo Erba CHNS elemental analyzer model EA1108.

MIR spectra (4000–200 cm^–1^) were recorded on KBr pellets with a Bruker EQUINOX 55 FT-spectrometer. FT-Raman spectra were recorded on a solid sample in a capillary tube, resolution ±4 cm^–1^, power 50 mW, Bruker model RFS100/S FT-spectrometer, operating with an excitation frequency of 1064 nm, Nd:YAG laser, and an indium–gallium–arsenide detector. Electronic spectra were recorded on organic solutions (solvent as specifically indicated) by a Varian Cary 5 Spectrophotometer. ^1^H NMR spectra were recorded at 300 K using a Varian Unity Inova 500 NMR spectrometer (Agilent Technologies, Santa Clara, CA). The residual signal of the deuterated solvents was used as standard and referenced to tetramethylsilane (TMS) (δ = 0.00 ppm).

### 4.4 Crystal Structure Determination

#### 4.4.1 X-ray Diffraction

X-ray data collections were performed on a Bruker Smart APEX II diffractometer for *^**i**^***Pr-1I**_**3**_ and **Me-1I**_**3**_ and an Oxford Diffraction Excalibur 3 diffractometer for **Bz-1I**_**3**_, **Ph-1CI**_**3**_, and **Ph-1** both equipped with a charge-coupled device (CCD) area detector and Mo Kα radiation (λ = 0.71073) at room temperature (**Bz-1I**_**3**_), 150 K (**Ph-1CI**_**3**_ and **Ph-1**) and 100 K (*^**i**^***Pr-1I**_**3**_ and **Me-1I**_**3**_) by the ω-scan method.

Data collection, data reduction, and absorption correction were performed using Bruker SMART, SAINT, and SADABS software^45^ (*^**i**^***Pr-1I**_**3**_ and **Me-1I**_**3**_) and CrysAlis CCD,^46^ CrysAlis RED,^47^ and ABSPACK^47^ software (**Bz-1I**_**3**_, **Ph-1CI**_**3**_, and **Ph-1**).

SIR2004^48^ and SHELXL programs^49^ were used for structure solution and refinement on *F*^2^ by full-matrix least-squares techniques. All non-hydrogen atoms were refined using anisotropic displacement parameters. The H atoms were located from different Fourier syntheses (*^**i**^***Pr-1I**_**3**_ and **Me-1I**_**3**_) or placed in geometrically calculated positions (**Bz-1I**_**3**_, **Ph-1CI**_**3**_, and **Ph-1**) and included in the refinement using a riding model in conjunction with a *U*_iso_(H) = 1.5 *U*_eq_(CH_3_, SH) or *U*_iso_(H) = 1.2 *U*_eq_(CH_2_) constraint.

The diagram was drawn using ORTEPIII program.^50^ Crystal data and structure determination results are summarized in Table 2.

**Table 2:** Crystal Data and Structure Refinement^a^

	**Me-1I_3_**	**Me-1I_3_*n***	***^i^*Pr-1I_3_**	**Bz-1I_3_**	**Ph-1CI_3_**	**Ph-1**
empirical formula	C_6_H_11_N_2_S_2_·I_3_	C_6_H_11_N_2_S_2_·I_3_	C_10_H_19_N_2_S_2_·I_3_	C_18_H_19_I_3_N_2_S_2_	C_16_H_13_I_3_N_2_S_2_	C_16_H_14_N_2_S_2_
formula weight	555.99	555.99	612.09	708.17	678.1	298.41
temp (K)	100(2)	100(2)	100(2)	293(2)	150(2)	150(2)
wavelength (Å)	0.71073	VIVALDI,^b^ white-beam	0.71073	0.71073	0.71073	0.71069
crystal system	monoclinic		triclinic	triclinic	monoclinic	triclinic
space group	*P*2_1_/*c*		*P*1̅	*P*1̅	*P*2_1_/*c*	*P*1̅
*a* (Å)	8.752(3)		9.5002(13)	10.2189(13)	22.6070(8)	7.3613(9)
*b* (Å)	11.168(4)		9.8785(13)	11.3571(15)	7.0043(3)	9.8623(14)
*c* (Å)	14.252(5)		11.5160(16)	11.5223(17)	12.3213(5)	10.7827(13)
α (deg)			71.473(2)	119.4670(15)		80.148(11)
β (deg)	93.876(4)		72.992(2)	97.7100(12)	90.851(3)	79.449(10)
γ (deg)			61.4250(10)	90.0390(11)		72.055(11)
*V* (Å^3^)	1389.9(8)		887.0(2)	1150.3(3)	1950.82(13)	726.62(17)
*Z*	4		2	2	4	2
ρ_calc_ (g cm^–3^)	2.657		2.292	2.045	2.309	1.364
μ (mm^–1^)	7.013		5.506	4.262	5.021	0.357
*F*(000)	1008		568	664	1256	312
θ range for data collection (deg)	2.3–31.4	3.3–38.7	2.4–31.5	4.12–28.78	3.81–29.08	4.135–32.359
index ranges	–12 ≤ *h* ≤ 12	–8 ≤ *h* ≤ 8	–13 ≤ *h* ≤ 13	–11 ≤ *h* ≤ 13	–29 ≤ *h* ≤ 30	–10 ≤ *h* ≤ 10
	–15 ≤ *k* ≤ 16	13 ≤ *k* ≤ 13	–13 ≤ *k* ≤ 14	–15 ≤ *k* ≤ 14	–6 ≤ *k* ≤ 9	–14 ≤ *k* ≤ 14
	–20 ≤ *l* ≤ 20	–17 ≤ *l* ≤ 16	–16 ≤ *l* ≤ 16	–12 ≤ *l* ≤ 14	–15 ≤ *l* ≤ 16	–15 ≤ *l* ≤ 13
reflections collected	21 585	4727	13 351	8435	16 137	6578
independent reflections	4363 [*R*_int_ = 0.0257]	4727	5364 [*R*_int_ = 0.0148]	5134 [*R*_int_ = 0.0325]	4601 [*R*_int_ = 0.0454]	4586 [*R*_int_ = 0.0363]
completeness to θ = 25.0°	99.8%		98.6%	99.3%	99.4%	99.2%
data/restraints/parameters	4363/0/151	4727/0/97	5364/0/211	5134/0/233	4601/0/249	4586/0/193
goodness-of-fit on *F*^2^	1.113	1.081	1.277	0.732	0.950	1.048
final *R* indices [*I* > 2σ(*I*)]	*R*_1_ = 0.0190	*R*_1_ = 0.2459	*R*_1_ = 0.0193	*R*_*1*_ = 0.0396	*R*_*1*_ = 0.0325	*R*_*1*_ = 0.0596
	w**R**_2_ = 0.0477	w**R**_2_ = 0.4291	w**R**_2_ = 0.0491	w**R**_2_ = 0.0808	w**R**_2_ = 0.0587	w**R**_2_ = 0.1425
*R* indices (all data)	*R*_1_ = 0.0224	*R*_1_ = 0.4040	*R*_1_ = 0.0202	*R*_*1*_ = 0.115	*R*_*1*_ = 0.0597	*R*_*1*_ = 0.0894
	w**R**_2_ = 0.0503	w**R**_2_ = 0.5296	w**R**_2_ = 0.0499	w**R**_2_ = 0.0976	w**R**_2_ = 0.0636	w**R**_2_ = 0.1700
largest diff. peak and hole (e Å^–3^)	0.896 and −0.856	1.424 and −0.993	0.622 and −1.620	0.47 and −0.687	0.762 and −0.660	0.790 and −0.648

a *n*, neutron diffraction structure.

b The wavelength-dependent intensities were then normalized to a constant incident wavelength.

#### 4.4.2 Neutron Diffraction

Neutron diffraction experiments on **Me-1I**_**3**_ complexes (**Me-1I**_**3**_***n***) were carried out using the very-intense vertical-axis Laue diffractometer (VIVALDI)^51,52^ at the Institut Laue-Langevin, Grenoble, with a white neutron beam covering wavelengths from 0.8 to 5.2 Å. The crystal (dimensions) was glued on a vanadium pin using an epoxy resin, mounted on the diffractometer, and cooled down to 100 K in an orange He flow cryostat.

To ensure full coverage of reciprocal space, a total of 11 Laue diffraction patterns were collected at 15° intervals in rotation about the vertical axis perpendicular to the incident beam, with an exposure time for each frame of around 3.8 h.

The Laue patterns were indexed and integrated using the LAUEGEN software^53^ suite, and wavelength normalization was carried out using the LAUENORM program.^54^ Correction for absorption was deemed unnecessary due to the small crystal dimensions.

Since the indexing of a Laue pattern provides only relative unit cell dimension, the absolute unit cell parameters were determined from X-ray, working at the same temperature as the neutron analysis. Data were refined by full-matrix least-squares starting from existing X-ray models based only on the atomic coordinates for the heavy atoms while all hydrogen atoms were located from Fourier difference maps.

Considering very limited sample dimensions, these collected neutron data were of sufficiently good quality to find hydrogen positions and confirm the S–H assignment. However, only individual isotropic factors were used in the refinement.

The collected data were of poor quality but good enough to find hydrogen positions and confirm the S–H assignment. However, only individual isotropic factors were used in the refinement.

### 4.5 Computational Details

All of the compounds were optimized with the Gaussian16 suite of program^55^ using the hybrid density functional B97D.^56^ All of the free energies, derived after the calculations of the vibrational frequencies, refer to a temperature of 298 K. All of the calculations were based on the CPCM model^57,58^ for the chloroform solvent. The basis set 6-31G inclusive of polarization functions was used for all species, while for iodine the Stuttgart/Dresden (SDD) pseudo-potential^59^ was employed. The coordinates of the optimized structures and their energetic parameters are reported in Section S6, Supporting Information.

## Supporting Information Available

Details on the characterization of the samples; ESI-MS (Section S1); vibrational spectroscopy (Section S3); NMR (Section S4); crystallography (Sections S5 and S6); details and figures on DFT calculations (Section S2); and radical trapping experiments (Section S7) (PDF)
