# Supplementary material for: Halogen-Bonding-Mediated Radical Reactions: The Unexpected Behavior of Piperazine-Based Dithiooxamide Ligands in the Presence of Diiodine
Source: Inorg Chem. 2023 Jan 5;62(2):694–705. doi: 10.1021/acs.inorgchem.2c02340 (PMC9846695; doi:10.1021/acs.inorgchem.2c02340)
Supplement: Supplementary file 1 — ic2c02340_si_001.pdf [file ic2c02340_si_001.pdf]

Supporting Information

Halogen-bonding mediated radical reactions: the unexpected behavior of piperazine-based dithiooxamide ligands in the presence of diiodine.

Silvia Rizzato,[a] Gabriele Manca,[b] Marie-Hélène Lemée,[c] Luciano Marchiò,[d] Flaminia Cesare Marincola,[e] Annalisa Guerri,[f] Andrea Ienco,[b]* Angela Serpe[g,h]* and Paola Deplano.[e,g]

[a]Dipartimento di Chimica, Università degli Studi di Milano, Via Golgi 19, I-20133 Milano, Italy.
[b]Istituto di Chimica dei Composti Organometallici ICCOM-CNR, Via Madonna del Piano 10, I-50019 Sesto Fiorentino (Florence), Italy.
[c]Institut Laue-Langevin, 71 avenue des Martyrs, CS 20156, 38042 Grenoble Cedex 9, France.
[d]Dipartimento di Chimica, Scienze della Vita e della Sostenibilità Ambientale, Università di Parma, 43124 Parma, Italy.
[e]Dipartimento di Scienze Chimiche e Geologiche, Università di Cagliari, 09042 Monserrato, Cagliari, Italy.
[f]Dipartimento di Chimica "Ugo Schiff", Università di Firenze, Via della Lastruccia 3, 50019 Sesto Fiorentino, Firenze, Italy.
[g]Dipartimento di Ingegneria Civile, Ambientale e Architettura (DICAAR) and Research Unit of INSTM, Università di Cagliari, I-09042 Monserrato (Cagliari), Italy.
[h]Istituto di Geologia Ambientale e Geoingegneria del Consiglio Nazionale delle Ricerche (IGAG-CNR), Piazza d'Armi, 09123 Cagliari, Italy.

Corresponding authors email address: AS, serpe@unica.it; AI, andrea.ienco@iccom.cnr.it.

Table of contents:

S1. ESI-MS of Me-1I$_3$: experimental details and spectrum.

S2. DFT calculations

S3. Vibrational spectroscopy investigation on R-1HI$_3$ salts

S4. NMR spectra

S5. Crystallography

S6. List of Cartesian coordinates and energies for the optimized structures (coordinates in Å and Energies in au)

S7. Radical trapping experiments

References

S1. ESI-MS of Me-1I$_3$: experimental details and spectrum.
S1.1. Experimental

The ESI-MS spectrum of Me-1HI$_3$ was recorded with a Micromass ZMD spectrometer, injection flow-rate = 20 µl/min; desolvation temperature = 150 °C; source block temperature = 80 °C; cone and desolvation gas flow-rates = 1.6 and 8 l/min, respectively; capillary = 3.0 KV; cone = 30 V; extractor = 3 V. Spectra were registered with a scansion time of 6 s.

The spectra of the other R-1HI$_3$ salts and of Ph-1CI$_3$ were carried out by using a Waters Acquity Ultra Performance LC with Waters Acquity SQ Detector and with ESI interface. Source Temperature (°C) 150; desolvation temperature (°C) 300; cone gas flow (L/Hr) 100; desolvation gas flow (L/Hr) 480; injection flow-rate= 0.2 ml/min; capillary voltage (kV) 3; cone voltage (V) 20/30).

All the mixtures were prepared in methanol and analyzed in negative and positive ionization mode by direct perfusion in ESI-MS interface.

S1.2. ESI-MS spectrum and description

I_3^- anion is identified by m/z 387 peak and the presence of the **Me-1H$^+$** specie is demonstrated from the m/z 175 peak. In the case of the non-protonated **Me-1$^{\bullet+}$** moiety, a peak at m/z 174 and/or a peak at m/z 86, corresponding to a **Me-1H^{2+}** specie resulting by the protonation in solution of the **Me-1$^{\bullet+}$**, are expected. None of them are instead found in the spectrum, furtherly supporting the presence of the protonated form in the sample. The ESI-MS characterization of the other **R-1H** salts, carried out several years later, confirmed this finding (see section 4.2 of the manuscript).

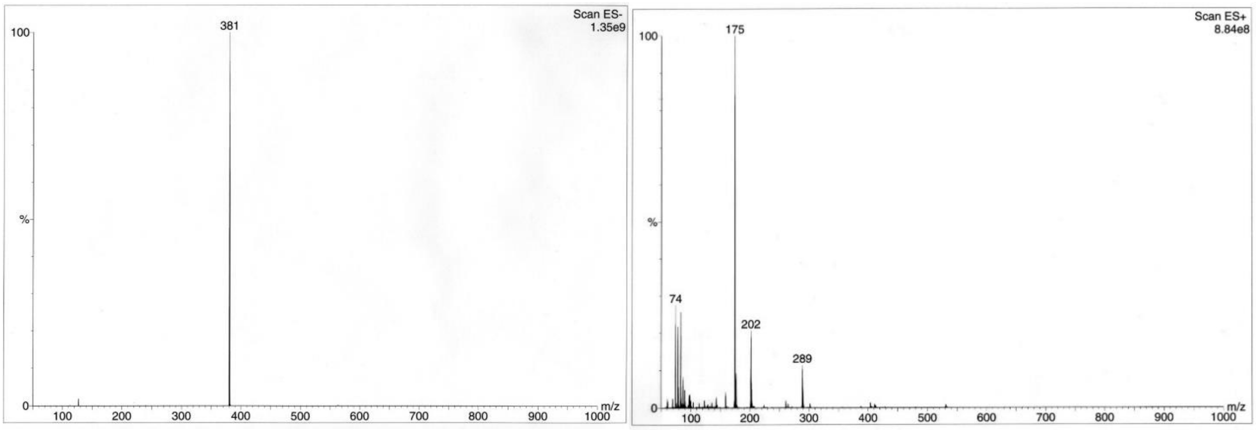

Figure S1. Electrospray-Mass Spectroscopy spectrum of Me-1HI$_3$ in MeOH solution.

S2. DFT calculations

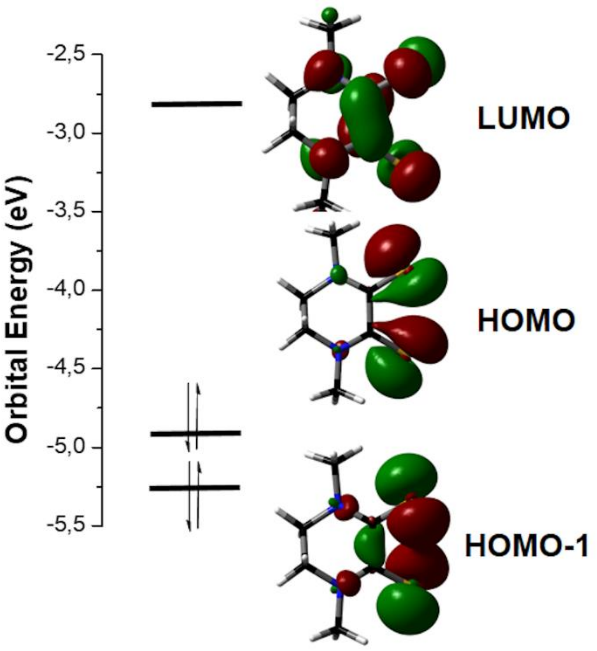

Figure S2. Frontier orbitals of the calculated **Me-1**. Given the shorter S-S distance (3.42 Å), the HOMO orbital has a S-S antibonding character.

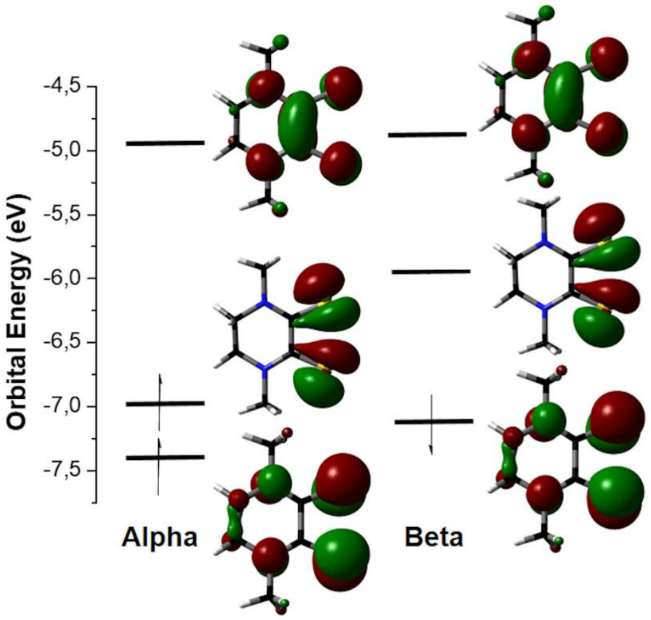

Figure S3. Frontier orbitals of the calculated **Me-1**$^{\cdot+}$. The alpha SOMO is sulfur-sulfur antibonding, while the corresponding beta orbital is empty. The result is the weakening the antibonding character between the two sulfur atoms and the formation of the S-S half bond with an S-S calculated distance of 2.83Å.

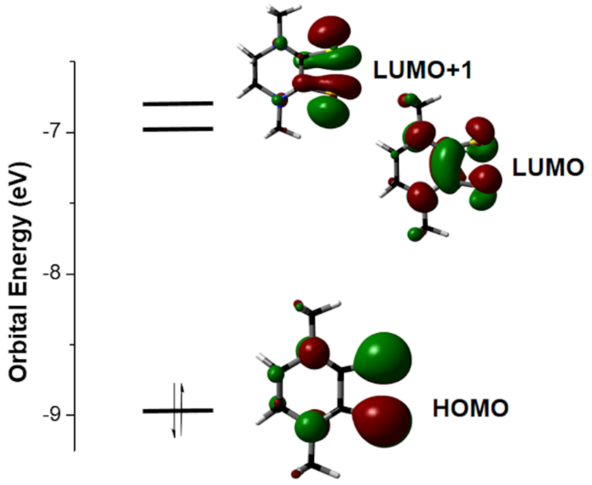

Figure S4. Frontier orbitals of the calculated **Me-1**$^{2+}$. Given the shorter S-S distance (2.22 Å), the S-S antibonding orbital is now fully depopulated and it became the LUMO+1.

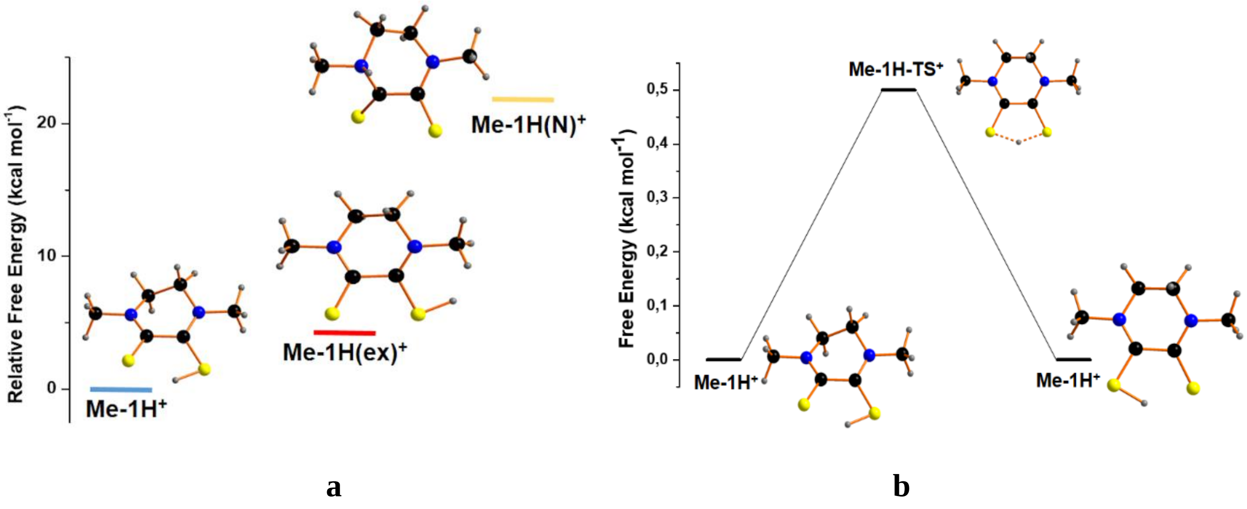

Figure S5. a) Calculated energy for the **Me-1H**$^+$ isomers: S-protonated isomer with intramolecular hydrogen bond (**Me-1H**$^+$); S-protonated isomer without intramolecular hydrogen bond; N-protonated isomer (**Me-1H(N)**$^+$). b) Free energy profile for the H$^+$ transfer from S1 to S2 in **Me-1H**$^+$.

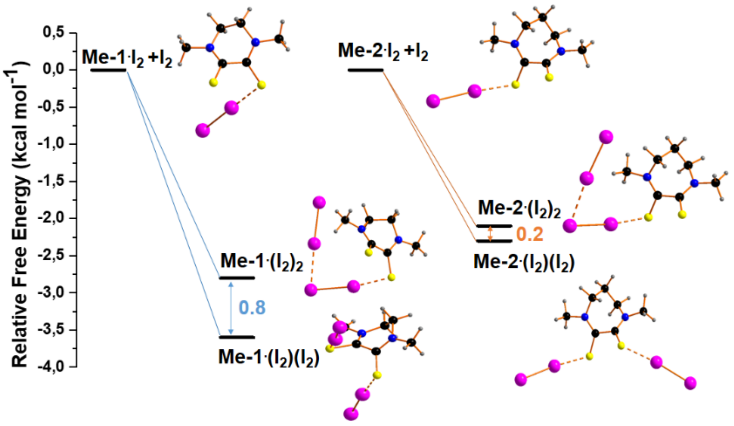

Figure S6. Free energy difference of the two possible isomers for the interaction of the second I_2 molecule to **Me-1·I**$_2$ and **Me-2·I**$_2$. In both cases **Me-1·I**$_2$ and **Me-2·I**$_2$ are taken as reference.

S3. Vibrational spectroscopy investigation on R-1HI$_3$ salts

The comparison of the υ(CN) IR and Raman bands of the free ligands to the ones of the corresponding salts can easily shed light on the weight of the resonance structures on the hybrid, so the corresponding thiolate character:

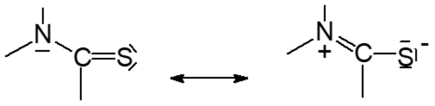

(a) (b)

Although the C-S bond should be the most affected by the coordination, the υCS, being a weak vibration and often coupled with other modes, is difficult to be unambiguously identified. The υ(CN) band is much easier identified and it invariably shows a shift towards higher frequencies with the formation of the halogen bonded complex due to the higher contribution of structure (b) to the resonance hybrid as summarized in table S1.

Compound		ν(CN)
**Me-2**	IR	1493vs-br
	R	1495w
**Me-2·2I$_2$**	IR	1530vs
	R	1532w
**Me-1**	IR	1503vs
	R	1504vw
**Me-1HI$_3$**	IR	1518s-br
	R	1562mw
**iPr-1**	IR	1471vs
	R	1468vw
**iPr-1HI$_3$**	IR	1505s
	R	-
**Bz-1**	IR	1484vs
	R	-
**Bz-1HI$_3$**	IR	1493s
	R	-

Table S1. Comparison between IR and Raman absorptions (cm^{-1}) tentatively assigned to **R-1** and **R-2** halogen bonded complexes and their precursors. As expected, the relative intensity of the peaks is reversed in IR *vs* Raman spectra.

S4. NMR spectra

For the sake of solubility and solvent of use in metal leaching, the NMR spectrum of the **Me-1I$_3$** was collected in CD_3CN. The spectrum of the salt was compared with the one of the free ligand **Me-1** in the same solvent. Figure S7 summarizes the cited spectra.

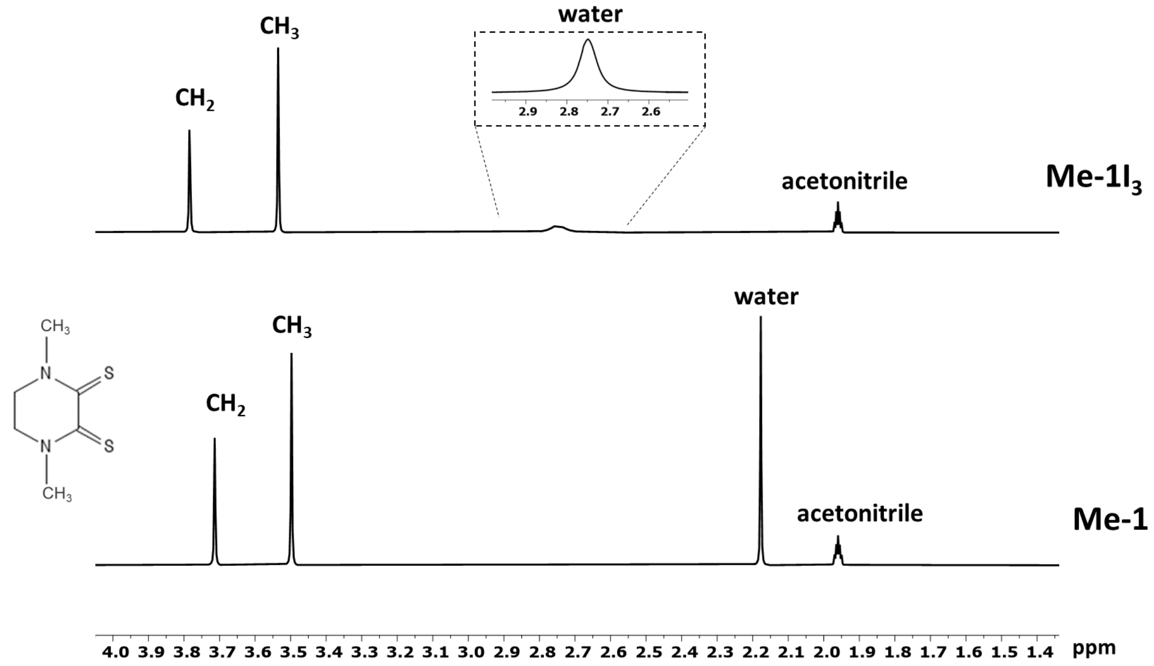

Figure S7. ^{1}H NMR spectra (CD_3CN) of **Me-1** (bottom) and **Me-1I₃** (top).

S5. Crystallography

S5.1 X-Ray diffraction

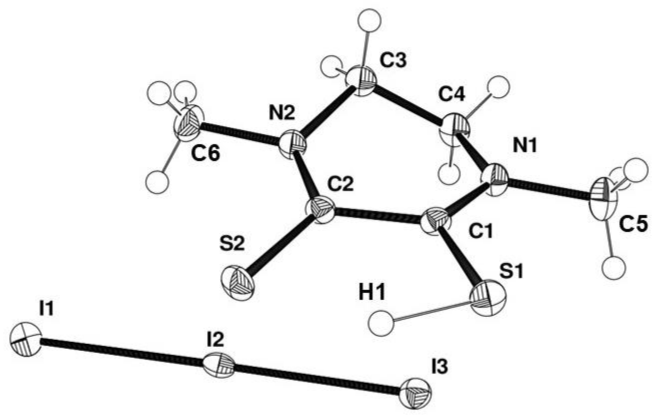

Figure S8. Atom labelling scheme for **Me-1I$_3$**.

Table S2. Bond lengths [Å] and angles [°] for **Me-1I$_3$.**

C(1)-N(1)	1.308(3)	C(5)-N(1)	1.474(3)
C(1)-C(2)	1.523(3)	C(5)-H(5B)	0.83(3)
C(1)-S(1)	1.709(2)	C(5)-H(5C)	0.94(3)
C(2)-N(2)	1.325(3)	C(5)-H(5A)	0.93(3)
C(2)-S(2)	1.672(2)	C(6)-N(2)	1.462(3)

C(3)-N(2)	1.470(3)	C(6)-H(6B)	0.83(3)
C(3)-C(4)	1.504(3)	C(6)-H(6A)	0.94(3)
C(3)-H(3A)	0.96(3)	C(6)-H(6C)	0.92(3)
C(3)-H(3B)	0.95(3)	S(1)-H(1)	1.40(3)
C(4)-N(1)	1.474(3)	I(1)-I(2)	2.9317(8)
C(4)-H(4A)	0.88(3)	I(2)-I(3)	2.9182(8)
C(4)-H(4B)	0.91(3)		
N(1)-C(1)-C(2)	120.22(16)	N(1)-C(5)-H(5C)	109.4(19)
N(1)-C(1)-S(1)	120.47(15)	H(5B)-C(5)-H(5C)	105(3)
C(2)-C(1)-S(1)	119.28(13)	N(1)-C(5)-H(5A)	109.1(19)
N(2)-C(2)-C(1)	116.57(16)	H(5B)-C(5)-H(5A)	116(3)
N(2)-C(2)-S(2)	124.37(15)	H(5C)-C(5)-H(5A)	110(3)
C(1)-C(2)-S(2)	119.06(14)	N(2)-C(6)-H(6B)	109.9(19)
N(2)-C(3)-C(4)	111.32(16)	N(2)-C(6)-H(6A)	108.1(19)
N(2)-C(3)-H(3A)	106.2(17)	H(6B)-C(6)-H(6A)	116(3)
C(4)-C(3)-H(3A)	110.7(17)	N(2)-C(6)-H(6C)	107.3(19)
N(2)-C(3)-H(3B)	110.1(17)	H(6B)-C(6)-H(6C)	112(3)
C(4)-C(3)-H(3B)	111.2(16)	H(6A)-C(6)-H(6C)	103(3)
H(3A)-C(3)-H(3B)	107(2)	C(1)-N(1)-C(4)	121.72(17)
N(1)-C(4)-C(3)	110.92(16)	C(1)-N(1)-C(5)	120.73(18)
N(1)-C(4)-H(4A)	105.4(18)	C(4)-N(1)-C(5)	116.82(17)
C(3)-C(4)-H(4A)	113.0(17)	C(2)-N(2)-C(6)	120.47(18)
N(1)-C(4)-H(4B)	107.8(18)	C(2)-N(2)-C(3)	122.47(16)
C(3)-C(4)-H(4B)	110.8(18)	C(6)-N(2)-C(3)	115.96(17)
H(4A)-C(4)-H(4B)	109(2)	C(1)-S(1)-H(1)	88.0(12)
N(1)-C(5)-H(5B)	106(2)	I(3)-I(2)-I(1)	178.025(6)

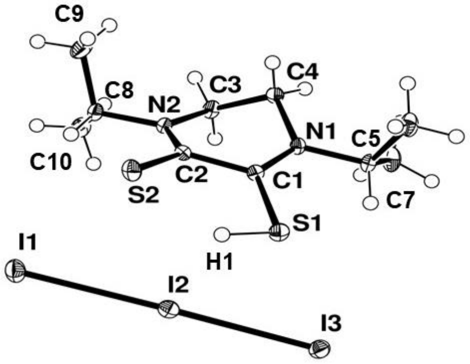

Figure S9. Atom labelling scheme for **^{i}Pr-1I$_3$**.

Table S3. Bond lengths [Å] and angles [°] for **^{i}Pr-1I$_3$.**

C(1)-N(1)	1.308(2)	C(6)-H(6C)	0.98(3)
C(1)-C(2)	1.527(3)	C(7)-H(7A)	0.98(3)
C(1)-S(1)	1.7113(19)	C(7)-H(7B)	0.93(3)
C(2)-N(2)	1.328(2)	C(7)-H(7C)	0.97(3)
C(2)-S(2)	1.6724(19)	C(8)-N(2)	1.485(2)
C(4)-N(1)	1.473(2)	C(8)-C(10)	1.516(3)
C(4)-C(3)	1.501(3)	C(8)-C(9)	1.523(3)
C(4)-H(4A)	0.95(3)	C(8)-H(8)	0.91(3)
C(4)-H(4B)	0.96(3)	C(9)-H(9A)	0.94(3)
C(3)-N(2)	1.467(2)	C(9)-H(9B)	0.95(3)
C(3)-H(3A)	0.94(3)	C(9)-H(9C)	0.99(3)
C(3)-H(3B)	0.94(3)	C(10)-H(10A)	1.01(3)
C(5)-N(1)	1.497(2)	C(10)-H(10B)	0.90(3)
C(5)-C(7)	1.520(3)	C(10)-H(10C)	0.95(3)
C(5)-C(6)	1.524(3)	S(1)-H(1)	1.22(3)
C(5)-H(5)	0.93(3)	I(2)-I(3)	2.9137(4)
C(6)-H(6A)	0.96(3)	I(2)-I(1)	2.9154(4)
C(6)-H(6B)	0.98(3)		
N(1)-C(1)-C(2)	119.80(16)	C(5)-C(7)-H(7B)	113(2)
N(1)-C(1)-S(1)	121.62(14)	H(7A)-C(7)-H(7B)	108(3)
C(2)-C(1)-S(1)	118.57(13)	C(5)-C(7)-H(7C)	109.2(19)
N(2)-C(2)-C(1)	115.88(16)	H(7A)-C(7)-H(7C)	106(3)
N(2)-C(2)-S(2)	125.11(15)	H(7B)-C(7)-H(7C)	110(3)
C(1)-C(2)-S(2)	118.95(13)	N(2)-C(8)-C(10)	109.58(16)
N(1)-C(4)-C(3)	108.31(16)	N(2)-C(8)-C(9)	110.81(16)
N(1)-C(4)-H(4A)	107.9(18)	C(10)-C(8)-C(9)	111.96(18)
C(3)-C(4)-H(4A)	109.7(18)	N(2)-C(8)-H(8)	105.6(19)
N(1)-C(4)-H(4B)	107.9(18)	C(10)-C(8)-H(8)	108.3(19)
C(3)-C(4)-H(4B)	111.7(18)	C(9)-C(8)-H(8)	110.4(19)
H(4A)-C(4)-H(4B)	111(3)	C(8)-C(9)-H(9A)	112.3(19)
N(2)-C(3)-C(4)	109.65(15)	C(8)-C(9)-H(9B)	106.3(19)
N(2)-C(3)-H(3A)	110.3(19)	H(9A)-C(9)-H(9B)	108(3)
C(4)-C(3)-H(3A)	111.7(19)	C(8)-C(9)-H(9C)	112.5(19)
N(2)-C(3)-H(3B)	107.4(18)	H(9A)-C(9)-H(9C)	103(3)
C(4)-C(3)-H(3B)	110.2(18)	H(9B)-C(9)-H(9C)	115(3)

H(3A)-C(3)-H(3B)	107(3)	C(8)-C(10)-H(10A)	107.5(18)
N(1)-C(5)-C(7)	108.80(16)	C(8)-C(10)-H(10B)	111(2)
N(1)-C(5)-C(6)	111.29(16)	H(10A)-C(10)-H(10B)	114(3)
C(7)-C(5)-C(6)	113.15(18)	C(8)-C(10)-H(10C)	107.0(19)
N(1)-C(5)-H(5)	105.8(19)	H(10A)-C(10)-H(10C)	107(3)
C(7)-C(5)-H(5)	111.6(18)	H(10B)-C(10)-H(10C)	109(3)
C(6)-C(5)-H(5)	105.9(19)	C(1)-N(1)-C(4)	117.74(16)
C(5)-C(6)-H(6A)	114.0(19)	C(1)-N(1)-C(5)	122.27(16)
C(5)-C(6)-H(6B)	112.3(19)	C(4)-N(1)-C(5)	119.69(15)
H(6A)-C(6)-H(6B)	107(3)	C(2)-N(2)-C(3)	120.83(16)
C(5)-C(6)-H(6C)	110.1(19)	C(2)-N(2)-C(8)	121.96(16)
H(6A)-C(6)-H(6C)	105(3)	C(3)-N(2)-C(8)	117.20(15)
H(6B)-C(6)-H(6C)	108(3)	C(1)-S(1)-H(1)	90.5(15)
C(5)-C(7)-H(7A)	110.2(19)	I(3)-I(2)-I(1)	177.915(6)

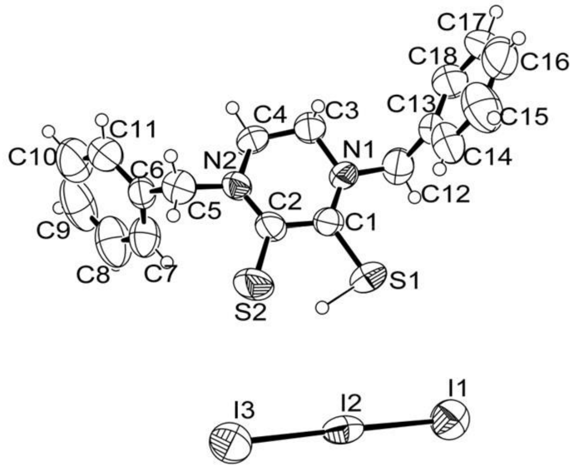

Figure S10. Atom labelling scheme for **Bz-1I$_3$**.

Table S4. Bond lengths [Å] and angles [°] for **Bz-1I$_3$.**

S(1)-C(1)	1.674(6)	C(8)-H(8)	0.93
S(1)-H(1)	1.60(6)	C(9)-C(10)	1.340(13)
S(2)-C(2)	1.669(5)	C(9)-H(9)	0.93
S(2)-H(1)	1.80(6)	C(10)-C(11)	1.367(12)
N(1)-C(1)	1.296(6)	C(10)-H(10)	0.93
N(1)-C(3)	1.449(7)	C(11)-H(11)	0.93
N(1)-C(12)	1.476(7)	C(12)-C(13)	1.475(8)
N(2)-C(2)	1.306(7)	C(12)-H(12A)	0.97
N(2)-C(4)	1.450(7)	C(12)-H(12B)	0.97

N(2)-C(5)	1.468(6)	C(13)-C(14)	1.352(8)
C(1)-C(2)	1.519(8)	C(13)-C(18)	1.358(8)
C(3)-C(5)	1.442(7)	C(14)-C(15)	1.368(10)
C(3)-H(3A)	0.99(4)	C(14)-H(14)	0.93
C(3)-H(3B)	0.99(4)	C(15)-C(16)	1.339(11)
C(4)-C(6)	1.490(8)	C(15)-H(15)	0.93
C(4)-H(4A)	0.87(4)	C(16)-C(17)	1.337(12)
C(4)-H(4B)	0.87(4)	C(16)-H(16)	0.93
C(5)-H(5A)	0.98(4)	C(17)-C(18)	1.399(12)
C(5)-H(5B)	0.98(4)	C(17)-H(17)	0.93
C(6)-C(7)	1.337(8)	C(18)-H(18)	0.93
C(6)-C(11)	1.362(9)	I(1)-I(2)	2.8972(7)
C(7)-C(8)	1.373(10)	I(2)-I(3)	2.8967(7)
C(7)-H(7)	0.93		
C(8)-C(9)	1.344(12)		
C(1)-S(1)-H(1)	87(2)	C(9)-C(8)-C(7)	120.1(10)
C(1)-N(1)-C(3)	121.4(5)	C(9)-C(8)-H(8)	120
C(1)-N(1)-C(12)	123.1(5)	C(7)-C(8)-H(8)	120
C(3)-N(1)-C(12)	115.1(4)	C(10)-C(9)-C(8)	119.7(10)
C(2)-N(2)-C(4)	122.8(5)	C(10)-C(9)-H(9)	120.1
C(2)-N(2)-C(5)	121.2(5)	C(8)-C(9)-H(9)	120.1
C(4)-N(2)-C(5)	115.6(5)	C(9)-C(10)-C(11)	119.6(10)
N(1)-C(1)-C(2)	118.9(5)	C(9)-C(10)-H(10)	120.2
N(1)-C(1)-S(1)	123.2(5)	C(11)-C(10)-H(10)	120.2
C(2)-C(1)-S(1)	117.9(4)	C(10)-C(11)-C(6)	121.6(9)
N(2)-C(2)-C(1)	118.4(5)	C(10)-C(11)-H(11)	119.2
N(2)-C(2)-S(2)	123.1-(5)	C(6)-C(11)-H(11)	119.2
C(1)-C(2)-S(2)	118.6(4)	C(13)-C(12)-N(1)	113.1(5)
C(5)-C(3)-N(1)	113.8(5)	C(13)-C(12)-H(12A)	109
C(5)-C(3)-H(3A)	108.8	N(1)-C(12)-H(12A)	109
N(1)-C(3)-H(3A)	108.8	C(13)-C(12)-H(12B)	109
C(5)-C(3)-H(3B)	108.8	N(1)-C(12)-H(12B)	109
N(1)-C(3)-H(3B)	108.8	H(12A)-C(12)-H(12B)	107.8
H(3A)-C(3)-H(3B)	107.7	C(14)-C(13)-C(18)	118.7(7)
N(2)-C(4)-C(6)	113.3(5)	C(14)-C(13)-C(12)	118.5(6)
N(2)-C(4)-H(4A)	108.9	C(18)-C(13)-C(12)	122.8(7)
C(6)-C(4)-H(4A)	108.9	C(13)-C(14)-C(15)	119.8(7)

N(2)-C(4)-H(4B)	108.9	C(13)-C(14)-H(14)	120.1
C(6)-C(4)-H(4B)	108.9	C(15)-C(14)-H(14)	120.1
H(4A)-C(4)-H(4B)	107.7	C(16)-C(15)-C(14)	122.1(9)
C(3)-C(5)-N(2)	112.7(5)	C(16)-C(15)-H(15)	119
C(3)-C(5)-H(5A)	109	C(14)-C(15)-H(15)	119
N(2)-C(5)-H(5A)	109	C(17)-C(16)-C(15)	119.1(11)
C(3)-C(5)-H(5B)	109	C(17)-C(16)-H(16)	120.4
N(2)-C(5)-H(5B)	109	C(15)-C(16)-H(16)	120.4
H(5A)-C(5)-H(5B)	107.8	C(16)-C(17)-C(18)	119.9(9)
C(7)-C(6)-C(11)	117.6(7)	C(16)-C(17)-H(17)	120.1
C(7)-C(6)-C(4)	119.8(6)	C(18)-C(17)-H(17)	120.1
C(11)-C(6)-C(4)	122.6(7)	C(13)-C(18)-C(17)	120.4(8)
C(6)-C(7)-C(8)	121.4(8)	C(13)-C(18)-H(18)	119.8
C(6)-C(7)-H(7)	119.3	C(17)-C(18)-H(18)	119.8
C(8)-C(7)-H(7)	119.3	I(3)-I(2)-I(1)	178.925(19)

The **Ph-1CI$_3$** salt crystallize in $P_{21/c}$ and in the asymmetric unit, one I_3^- and one **Ph-1C$^+$** cation are present as shown in Figure S11.

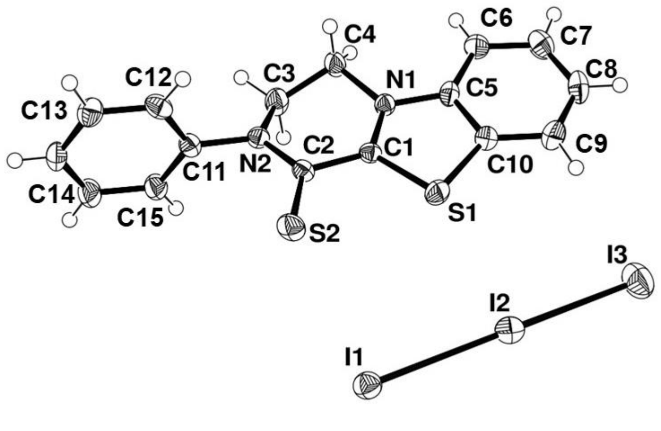

Figure S11. Atom labelling scheme for **Ph-1CI$_3$**.

The I_3^- anion is asymmetric. The I1-I2 distance (2.9683(4) Å) is longer than I2-I3 one (2.8401(4) Å) while for **Me-1I$_3$**, **^{i}PrBz-1I$_3$**, **Bz-1I$_3$** the two distances are of the same length. In the latter structures, I_3^- anions are located symmetrically respect the sulphur atoms of the cations. For **Ph-1CI$_3$** salt, two iodine atoms (I1 and I2) are much closer to S2 and S1 than I3 atom and this geometrical arrangement could explain the asymmetry of the anion. In the **Ph-1C$^+$** cation, the C1-C2 distance (1.470(5) Å) is shorter than the corresponding distance in **Me-1$^+$**, **^{i}PrBz-1$^+$**, **Bz-1$^+$**. The N1-C1 distance is 1.311(5) Å indicating a double bond character for the N1-C1 bond and the positive charge of the cation is mainly located on N1. Also, a partial electron delocalization is present on N1-C5 bond being the distance 1.386(5) Å. For N2, the shortest distance is with C2 (1.337(5) Å) and this bond distance is also shorter than the corresponding bond in **Me-1$^+$**, **^{i}PrBz-1$^+$**, **Bz-1$^+$** cations but close to the value found for **Ph-1** molecule (see below). The other two bond distance of N2 (N2-C3 1.337(5) Å and N2-

C11 1.441(5) Å) are longer. Thus, confirming a partial electron delocalization of the N2 lone pair on the C2 atom. The S1-C1 and the S1-C10 distances (1.700(4) Å, 1.732(4) Å respectively) are in the range for C*sp*^{2}-S distances [S1] and the S2-C2 distance is slightly shorter than expected for C*sp*^{2}=S distances. [S1]

Finally, all the atoms of benzothiazolium group lies on a common plane with the largest deviation of 0.031(4) Å for C10. S2 and N2 atoms are instead 0.448(2) Å and 0.068(4) Å over and below respectively the plane of benzothiazolium group. The dihedral angle S2-C2-N2-C11 are 86.7(5) ° showing that the phenyl ring is almost perpendicular to S2-C2-N2 plane.

Table S5. Bond lengths [Å] and angles [°] for **Ph-1CI$_3$**.

S(1)-C(1)	1.700(4)	C(7)-C(8)	1.378(7)
S(1)-C(10)	1.732(4)	C(7)-H(7)	0.91(5)
S(2)-C(2)	1.648(4)	C(8)-C(9)	1.359(7)
N(1)-C(1)	1.311(5)	C(8)-H(8)	0.87(5)
N(1)-C(5)	1.386(5)	C(9)-C(10)	1.392(6)
N(1)-C(4)	1.478(5)	C(9)-H(9)	0.92(5)
N(2)-C(2)	1.337(5)	C(11)-C(12)	1.369(6)
N(2)-C(11)	1.441(5)	C(11)-C(16)	1.379(6)
N(2)-C(3)	1.483(5)	C(12)-C(13)	1.389(6)
C(1)-C(2)	1.470(5)	C(12)-H(12)	0.91(4)
C(3)-C(4)	1.487(6)	C(13)-C(14)	1.369(6)
C(3)-H(3A)	0.97(5)	C(13)-H(13)	0.88(5)
C(3)-H(3B)	1.09(5)	C(14)-C(15)	1.375(6)
C(4)-H(4A)	1.03(5)	C(14)-H(14)	0.84(4)
C(4)-H(4B)	0.98(5)	C(15)-C(16)	1.375(6)
C(5)-C(6)	1.391(6)	C(15)-H(15)	0.89(4)
C(5)-C(10)	1.393(6)	C(16)-H(16)	0.81(5)
C(6)-C(7)	1.376(6)	I(1)-I(2)	2.9683(4)
C(6)-H(6)	0.87(5)	I(2)-I(3)	2.8401(4)
C(1)-S(1)-C(10)	90.2(2)	C(6)-C(7)-C(8)	121.3(5)
C(1)-N(1)-C(5)	114.7(3)	C(6)-C(7)-H(7)	111(3)
C(1)-N(1)-C(4)	120.5(3)	C(8)-C(7)-H(7)	127(3)
C(5)-N(1)-C(4)	124.5(3)	C(9)-C(8)-C(7)	122.6(5)
C(2)-N(2)-C(11)	121.6(3)	C(9)-C(8)-H(8)	118(3)
C(2)-N(2)-C(3)	120.5(3)	C(7)-C(8)-H(8)	119(3)
C(11)-N(2)-C(3)	117.2(3)	C(8)-C(9)-C(10)	117.4(5)
N(1)-C(1)-C(2)	124.5(3)	C(8)-C(9)-H(9)	128(3)
N(1)-C(1)-S(1)	113.4(3)	C(10)-C(9)-H(9)	115(3)

C(2)-C(1)-S(1)	122.0(3)	C(9)-C(10)-C(5)	120.2(4)
N(2)-C(2)-C(1)	114.4(3)	C(9)-C(10)-S(1)	128.9(4)
N(2)-C(2)-S(2)	127.3(3)	C(5)-C(10)-S(1)	110.9(3)
C(1)-C(2)-S(2)	118.2(3)	C(12)-C(11)-C(16)	120.9(4)
N(2)-C(3)-C(4)	112.3(4)	C(12)-C(11)-N(2)	120.3(4)
N(2)-C(3)-H(3A)	108(3)	C(16)-C(11)-N(2)	118.7(4)
C(4)-C(3)-H(3A)	114(3)	C(11)-C(12)-C(13)	119.8(4)
N(2)-C(3)-H(3B)	104(3)	C(11)-C(12)-H(12)	120(3)
C(4)-C(3)-H(3B)	111(2)	C(13)-C(12)-H(12)	120(3)
H(3A)-C(3)-H(3B)	107(4)	C(14)-C(13)-C(12)	119.2(5)
N(1)-C(4)-C(3)	108.9(4)	C(14)-C(13)-H(13)	122(3)
N(1)-C(4)-H(4A)	105(3)	C(12)-C(13)-H(13)	119(3)
C(3)-C(4)-H(4A)	112(3)	C(13)-C(14)-C(15)	120.8(4)
N(1)-C(4)-H(4B)	107(3)	C(13)-C(14)-H(14)	117(3)
C(3)-C(4)-H(4B)	109(3)	C(15)-C(14)-H(14)	122(3)
H(4A)-C(4)-H(4B)	114(4)	C(16)-C(15)-C(14)	120.2(4)
N(1)-C(5)-C(6)	127.4(4)	C(16)-C(15)-H(15)	116(3)
N(1)-C(5)-C(10)	110.8(4)	C(14)-C(15)-H(15)	123(3)
C(6)-C(5)-C(10)	121.8(4)	C(15)-C(16)-C(11)	119.1(4)
C(7)-C(6)-C(5)	116.7(4)	C(15)-C(16)-H(16)	126(3)
C(7)-C(6)-H(6)	119(3)	C(11)-C(16)-H(16)	114(3)
C(5)-C(6)-H(6)	124(3)	I(3)-I(2)-I(1)	176.990(14)

Ph-1 crystallizes in *P-1* space group. The asymmetric unit consists of one molecule of **Ph-1** and it is shown in Figure S12.

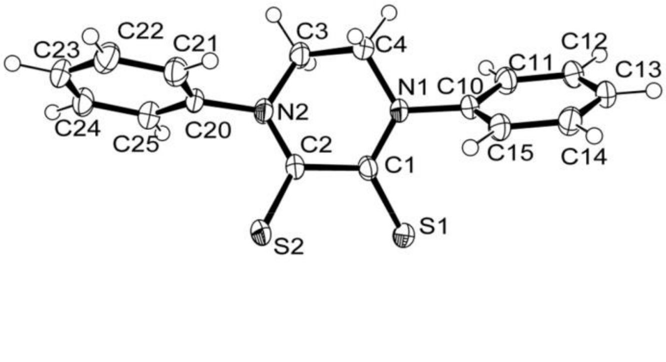

Figure S12. Atom labelling scheme for **Ph-1**.

The C1-C2 distance is 1.522(3) Å as expected for single C-C bond.[S1] The C1-S1 and C2-S2 are respectively 1.656(2) Å and 1.655(2) Å in the expected range for (X)2-C=S (X=C, N, O, S) systems.[S1] The dihedral angle S1-C1-C2-S2 is 36.8(2) °. For comparison, in **Me-1** [S2] and **^{i}Pr-1** [S3] the same angle

is 36.3 and 36.8 respectively. The C1-N1 and C2-N2 distances are 1.346(3) and 1.335(3) respectively shorter than the distances with the C_{ipso} of the phenyl ring (C10-N1 1.440(3); C20-N2 1.445(3)) and with the other carbon atoms of the piperazine ring (C4-N1 1.472(3); C3-N2 1.473(3)). This indicates a partial delocalization of the nitrogen lone pairs with the C1 and C2 atoms. The same trend is found for **Me-1** [S2] and **^{i}Pr-1**.[S3] The dihedral angles S1-C1-N1-C10 and S2-C2-N2-C20 are -71.4(3) ° and -93.7(3) showing that the phenyl rings are almost perpendicular to the S1-C1-N1 and S2-C2-N2 planes.

Table S6. Bond lengths [Å] and angles [°] for **1-Ph**.

N(1)-C(1)	1.346(3)	C(12)-C(13)	1.384(3)
N(1)-C(10)	1.440(3)	C(12)-H(12)	1.07(3)
N(1)-C(4)	1.472(3)	C(13)-C(14)	1.385(3)
N(2)-C(2)	1.335(3)	C(13)-H(13)	0.95(3)
N(2)-C(20)	1.445(3)	C(14)-C(15)	1.385(3)
N(2)-C(3)	1.473(3)	C(14)-H(14)	0.97(3)
S(1)-C(1)	1.656(2)	C(15)-H(15)	1.03(3)
S(2)-C(2)	1.655(2)	C(20)-C(25)	1.380(3)
C(1)-C(2)	1.522(3)	C(20)-C(21)	1.387(3)
C(3)-C(4)	1.500(3)	C(21)-C(22)	1.391(3)
C(3)-H(3A)	1.00(2)	C(21)-H(21)	0.95(3)
C(3)-H(3B)	1.00(2)	C(22)-C(23)	1.379(4)
C(4)-H(4A)	0.99(2)	C(22)-H(22)	0.97(3)
C(4)-H(4B)	0.99(2)	C(23)-C(24)	1.380(4)
C(10)-C(11)	1.383(3)	C(23)-H(23)	0.98(3)
C(10)-C(15)	1.384(3)	C(24)-C(25)	1.390(3)
C(11)-C(12)	1.390(3)	C(24)-H(24)	0.97(3)
C(11)-H(11)	0.93(3)	C(25)-H(25)	0.95(3)
C(1)-N(1)-C(10)	121.82(18)	C(11)-C(10)-N(1)	120.6(2)
C(1)-N(1)-C(4)	121.48(19)	C(15)-C(10)-N(1)	117.8(2)
C(10)-N(1)-C(4)	116.30(18)	C(10)-C(11)-C(12)	118.8(2)
C(2)-N(2)-C(20)	122.24(19)	C(13)-C(12)-C(11)	120.2(2)
C(2)-N(2)-C(3)	120.14(19)	C(12)-C(13)-C(14)	120.4(2)
C(20)-N(2)-C(3)	117.15(17)	C(15)-C(14)-C(13)	119.8(2)
N(1)-C(1)-C(2)	114.73(18)	C(10)-C(15)-C(14)	119.4(2)
N(1)-C(1)-S(1)	123.49(17)	C(25)-C(20)-C(21)	121.1(2)
C(2)-C(1)-S(1)	121.75(16)	C(25)-C(20)-N(2)	120.1(2)
N(2)-C(2)-C(1)	116.36(19)	C(21)-C(20)-N(2)	118.7(2)
N(2)-C(2)-S(2)	124.43(17)	C(20)-C(21)-C(22)	119.2(2)
C(1)-C(2)-S(2)	119.05(15)	C(23)-C(22)-C(21)	120.1(2)

N(2)-C(3)-C(4)	108.1(2)	C(22)-C(23)-C(24)	120.2(2)
N(1)-C(4)-C(3)	109.0(2)	C(23)-C(24)-C(25)	120.5(2)
C(11)-C(10)-C(15)	121.4(2)	C(20)-C(25)-C(24)	119.0(2)

S4.2 Neutron Diffraction

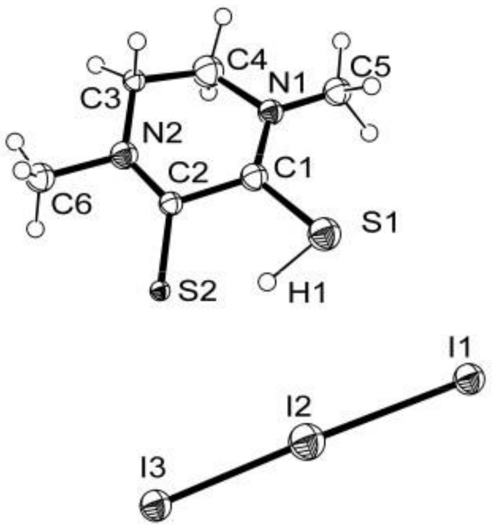

Figure S13. Atom labelling scheme for **Me-1I$_3$*n***.

Table S7. Bond lengths [Å] and angles [°] for **Me-1I$_3$*n*.**

S(1)-H(1)	1.34(6)	C(3)-C(4)	1.48(2)
S(1)-C(1)	1.73(5)	C(4)-H(4A)	1.05(5)
S(2)-C(2)	1.61(4)	C(4)-H(4B)	1.16(5)
N(1)-C(5)	1.30(2)	C(5)-H(5B)	1.09(8)
N(1)-C(1)	1.36(2)	C(5)-H(5C)	1.14(5)
N(1)-C(4)	1.53(2)	C(5)-H(5A)	1.15(6)
N(2)-C(2)	1.29(2)	C(6)-H(6B)	0.88(9)
N(2)-C(3)	1.41(2)	C(6)-H(6C)	1.03(6)
N(2)-C(6)	1.58(2)	C(6)-H(6A)	1.11(8)
C(1)-C(2)	1.58(2)	I(1)-I(2)	3.04(4)
C(3)-H(3A)	0.99(5)	I(2)-I(3)	2.83(4)
C(3)-H(3B)	1.34(5)		
H(1)-S(1)-C(1)	82(3)	H(4A)-C(4)-H(4B)	106(3)
C(5)-N(1)-C(1)	122.4(14)	H(4A)-C(4)-C(3)	112(2)
C(5)-N(1)-C(4)	119.9(15)	H(4B)-C(4)-C(3)	110(2)
C(1)-N(1)-C(4)	116.8(14)	H(4A)-C(4)-N(1)	107(3)
C(2)-N(2)-C(3)	129.5(14)	H(4B)-C(4)-N(1)	104(2)

C(2)-N(2)-C(6)	115.8(14)	C(3)-C(4)-N(1)	116.8(17)
C(3)-N(2)-C(6)	113.3(13)	H(5B)-C(5)-H(5C)	106(4)
N(1)-C(1)-C(2)	119.2(15)	H(5B)-C(5)-H(5A)	108(4)
N(1)-C(1)-S(1)	120(2)	H(5C)-C(5)-H(5A)	102(4)
C(2)-C(1)-S(1)	120(2)	H(5B)-C(5)-N(1)	114(4)
N(2)-C(2)-C(1)	115.2(14)	H(5C)-C(5)-N(1)	114(3)
N(2)-C(2)-S(2)	130(2)	H(5A)-C(5)-N(1)	111(3)
C(1)-C(2)-S(2)	114.1(19)	H(6B)-C(6)-H(6C)	118(5)
H(3A)-C(3)-H(3B)	114(3)	H(6B)-C(6)-H(6A)	99(6)
H(3A)-C(3)-N(2)	109(3)	H(6C)-C(6)-H(6A)	118(5)
H(3B)-C(3)-N(2)	108(3)	H(6B)-C(6)-N(2)	107(5)
H(3A)-C(3)-C(4)	113(3)	H(6C)-C(6)-N(2)	108(3)
H(3B)-C(3)-C(4)	103(2)	H(6A)-C(6)-N(2)	107(4)
N(2)-C(3)-C(4)	108.8(15)	I(3)-I(2)-I(1)	177.2(12)

S6. List of Cartesian coordinates and energies for the optimized structures (coordinates in Å and Energies in au)

Compound: **Me-1**

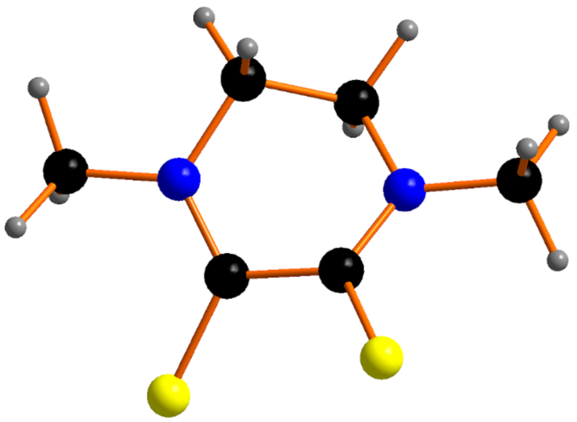

Cartesian Coordinates

C 0.767246 -0.325005 0.146500
C -0.738855 -0.316493 -0.145845
C -0.598669 2.061218 0.432244
C 0.654412 2.053594 -0.433919
H -1.216378 2.933146 0.188321
H 1.282108 2.918596 -0.190831
N -1.380415 0.842063 0.142342
N 1.422085 0.825806 -0.142836
S -1.507816 -1.662663 -0.799629
S 1.520702 -1.679276 0.801596
H 0.389162 2.092899 -1.503176
H -0.332977 2.098499 1.501463
C -2.841259 0.962980 0.042246
H -3.150995 1.026073 -1.012935
H -3.314201 0.080073 0.490387
H -3.148145 1.869553 0.577323
C 2.884225 0.929992 -0.042858
H 3.194673 0.990623 1.012258
H 3.346954 0.041219 -0.490064
H 3.201541 1.832401 -0.578888

Electronic Energy: -1140.3102383
Free Energy: -1140.1902890

Compound: **Me-1$^{\bullet+}$**

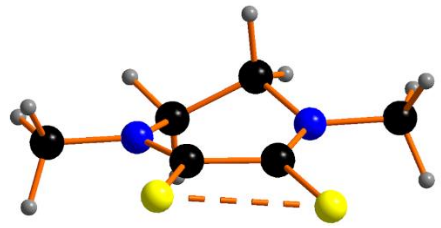

Cartesian Coordinates

C -1.227840 -0.737886 0.346565
C -0.956852 0.726561 0.462427
C -3.184893 1.126556 -0.415319
C -3.056818 -0.198836 -1.156419
H -3.834457 1.033501 0.468382
H -2.563946 -0.069521 -2.132060
N -1.854621 1.608530 0.037331
N -2.263375 -1.172350 -0.362987
C -1.636682 3.055906 0.218222
H -2.466142 3.595296 -0.248087
H -1.588044 3.289751 1.292190
H -0.688334 3.340232 -0.258219
C -2.496897 -2.616117 -0.553067
H -3.423759 -2.748872 -1.118479
H -1.651287 -3.056977 -1.101737
H -2.583499 -3.099979 0.429576
S 0.536765 1.090433 1.193951
S -0.091000 -1.710660 1.158804
H -4.055170 -0.620903 -1.311582
H -3.605453 1.877032 -1.092694

Electronic Energy: -1140.1098048
Free Energy: -1139.9896250

Compound: **Me-1^{2+}**

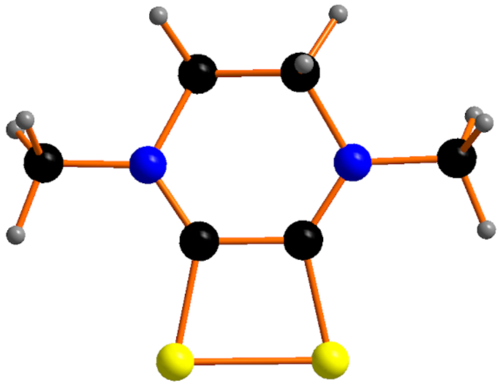

Cartesian Coordinates

C -1.248558 -0.722512 0.324588
C -0.989319 0.723534 0.453137

C -3.168851 1.141131 -0.430681
C -3.067707 -0.214670 -1.137823
H -3.824666 1.103690 0.450526
H -2.598048 -0.134156 -2.128255
N -1.835226 1.629569 0.060742
N -2.242410 -1.202760 -0.362474
C -1.644873 3.082468 0.222938
H -1.663612 3.538456 -0.775877
H -2.481564 3.466625 0.822191
H -0.691055 3.288682 0.719683
C -2.505216 -2.638852 -0.567501
H -3.503410 -2.859470 -0.166471
H -2.488220 -2.833029 -1.648597
H -1.746409 -3.241662 -0.057723
S 0.546814 0.755360 1.267628
S 0.086393 -1.418812 1.194743
H -4.072935 -0.635303 -1.246018
H -3.553434 1.883412 -1.137956

Electronic Energy: -1139.8501695
Free Energy: -1139.7274200

Compound: **Me-1H^{+}**

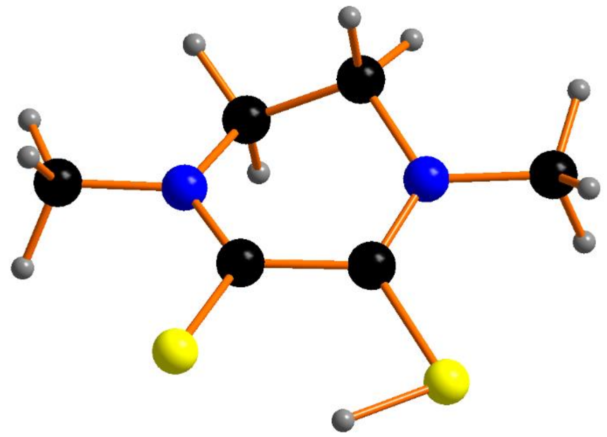

Cartesian Coordinates

S 1.633151 -1.745876 -0.289349
S -1.554517 -1.824800 0.276364
N 1.379266 0.864730 0.134858
N -1.428319 0.823517 -0.106109
C 0.749330 -0.287434 -0.027727
C -0.783189 -0.347389 0.036382
C -0.689440 2.063509 -0.424970
H -1.310705 2.919871 -0.144318
H -0.496662 2.101196 -1.508713
C 0.605832 2.110958 0.364776
H 0.424146 2.206190 1.445805
H 1.215809 2.953816 0.024342

C 2.855560 0.945033 0.181795
C -2.901436 0.859864 -0.125466
H -3.223652 1.888993 -0.310144
H -3.275920 0.194290 -0.917009
H -3.288553 0.507767 0.840824
H 3.264131 0.713843 -0.812244
H 3.140510 1.959473 0.474399
H 3.237144 0.222663 0.916763
H 0.420514 -2.441214 -0.157857

Electronic Energy: -1140.7309113
Free Energy: -1140.6015590

Compound: **Me-1H(N)$^{+}$**

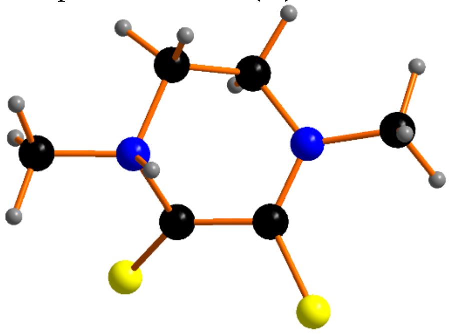

Cartesian Coordinates

C 0.746949 -0.448548 -0.130245
C -0.735747 -0.378802 -0.325286
C -0.625342 2.091016 0.234683
C 0.541442 1.871299 -0.744712
H -1.403010 2.700528 -0.237034
H 1.154816 2.776917 -0.766884
N -1.273686 0.740035 0.618389
N 1.374425 0.730746 -0.318748
S -1.695790 -1.215921 -1.313506
S 1.394751 -1.882305 0.431699
H 0.159446 1.684720 -1.758786
H -0.296351 2.553560 1.170278
C -2.773905 0.836290 0.746902
H -3.189809 1.074344 -0.236017
H -3.151265 -0.124686 1.107960
H -2.978089 1.635445 1.467193
C 2.821074 0.876319 -0.094081
H 3.039948 0.829796 0.983065
H 3.349755 0.055116 -0.596505
H 3.142194 1.838044 -0.506068
H -0.910904 0.489386 1.547701

Electronic Energy: -1140.6996538
Free Energy: -1140.566581

Compound: **Me-1H(ex)$^+$**

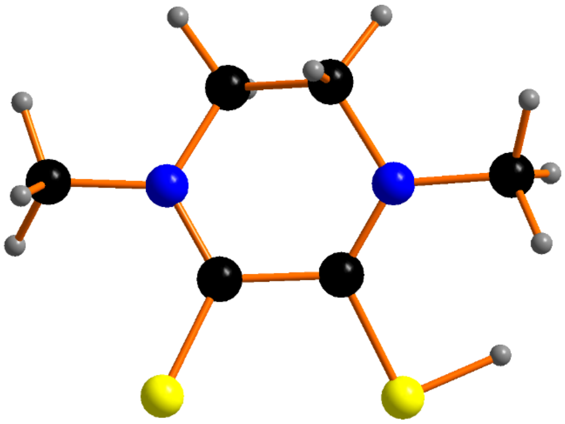

Cartesian Coordinates

S 1.513799 -1.821768 -0.431819
S -1.469740 -1.804823 0.511866
N 1.396658 0.832030 0.161863
N -1.410712 0.804958 -0.148331
C 0.749536 -0.300477 -0.059580
C -0.757929 -0.353690 0.068704
C -0.655471 2.020223 -0.511169
H -1.275391 2.895295 -0.291638
H -0.425275 2.007390 -1.588284
C 0.616629 2.092922 0.318125
H 0.405441 2.230747 1.388995
H 1.248151 2.914726 -0.037489
C 2.863132 0.938151 0.318197
C -2.880641 0.852917 -0.097986
H -3.207619 1.837427 -0.446576
H -3.296074 0.063491 -0.740228
H -3.223109 0.680493 0.932685
H 3.342379 0.959551 -0.671735
H 3.079350 1.871981 0.846903
H 3.234430 0.090277 0.906872
H 2.723955 -1.296920 -0.741974

Electronic Energy: -1140.7254557
Free Energy: -1140.594989

Compound: **Me-1H$^+$TS**

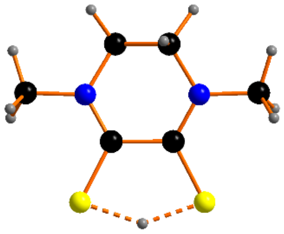

Cartesian Coordinates

S -1.541968 -1.807405 0.198700
S 1.545778 -1.803913 -0.213689
N -1.411575 0.855857 -0.072218
N 1.411426 0.857543 0.072665
C -0.767747 -0.303725 0.029225
C 0.769329 -0.302399 -0.035460
C 0.665769 2.104044 0.367091
H 1.265493 2.951754 0.020754
H 0.532003 2.178742 1.457183
C -0.667778 2.105147 -0.359447
H -0.534134 2.186335 -1.449090
H -1.268763 2.949950 -0.008223
C -2.887089 0.898572 -0.092488
C 2.886873 0.902355 0.093135
H 3.205145 1.939553 0.231505
H 3.259693 0.276778 0.917110
H 3.273381 0.512351 -0.858579
H -3.273041 0.502429 0.856913
H -3.206923 1.936080 -0.224804
H -3.258940 0.277264 -0.920123
H 0.002282 -2.334942 -0.009045

Electronic Energy: -1140.7289498
Free Energy: -1140.60076
Number of imaginary frequency 1

Compound: **Me-1·I_2**

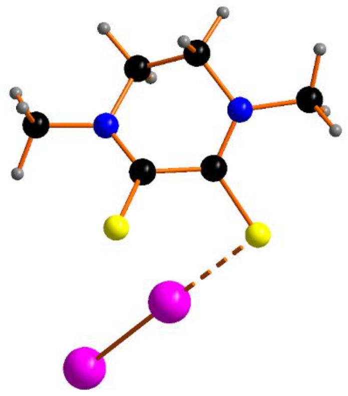

Cartesian Coordinates

C -4.220653 0.885204 -0.948974
C -4.462482 0.755643 0.551940
C -5.021741 3.115980 0.429667
C -3.746568 3.215226 -0.403358
H -5.918534 3.175573 -0.206126
H -2.855372 3.208497 0.244158
N -5.002820 1.822235 1.155513
N -3.708451 2.073223 -1.341521
C -5.444683 1.788134 2.561237

H -5.996620 2.710121 2.771026
H -6.094450 0.919378 2.724013
H -4.568464 1.710084 3.222682
C -3.326526 2.322211 -2.737585
H -2.670750 3.200433 -2.759873
H -2.798687 1.444881 -3.130774
H -4.220697 2.503712 -3.354994
S -4.086022 -0.669016 1.417834
S -4.670761 -0.357988 -1.976618
H -3.755816 4.141342 -0.988886
H -5.053684 3.918071 1.175075
I -1.533711 -1.310839 0.272652
I 1.177293 -1.902805 -0.721986

Electronic Energy: -1163.2482255
Free Energy: -1163.1385020

Compound: **Me-1·$(I_2)_2$**

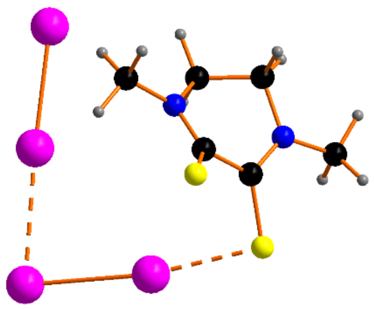

Cartesian Coordinates

C -4.020837 0.818422 -1.013096
C -4.542899 0.684316 0.413143
C -4.410466 3.103122 0.461520
C -3.038339 2.875685 -0.167685
H -5.139841 3.468382 -0.276995
H -2.305927 2.553677 0.588742
N -4.881110 1.812834 1.033142
N -3.171370 1.844547 -1.217332
C -5.572479 1.827787 2.337667
H -5.891647 2.854354 2.544039
H -6.446320 1.166940 2.302915
H -4.880823 1.479160 3.119108
C -2.535934 2.066366 -2.523136
H -1.697267 2.756251 -2.379651
H -2.173638 1.108063 -2.914736
H -3.263366 2.489916 -3.233449
S -4.709476 -0.851251 1.190594
S -4.615482 -0.207780 -2.195589
H -2.678465 3.799163 -0.632526
H -4.339741 3.820592 1.286292
I -2.176100 -1.693289 0.620170

I 0.790527 -2.129481 0.015477
I 0.760000 3.967279 -0.790856
I 0.844300 1.087543 -0.434658

Electronic Energy: -1186.1836668
Free Energy: -1186.0824000

Compound: **Me-1·(I₂)(I₂)** on two sulphur atoms

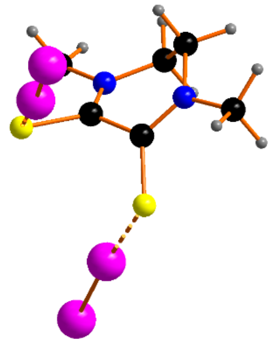

Cartesian Coordinates

C -4.238170 0.821166 -0.918040
C -4.481393 0.696326 0.579715
C -5.027006 3.052059 0.432602
C -3.742234 3.132874 -0.389959
H -5.918401 3.115721 -0.209841
H -2.851278 3.110359 0.255844
N -5.024976 1.765313 1.170389
N -3.718326 1.983032 -1.326624
C -5.455131 1.739401 2.578710
H -6.036128 2.646118 2.775875
H -6.074607 0.851468 2.754202
H -4.572108 1.700914 3.234824
C -3.290129 2.194914 -2.719761
H -2.728775 3.133681 -2.766288
H -2.652315 1.360484 -3.035822
H -4.173262 2.244655 -3.374969
S -4.027119 -0.708624 1.424987
S -4.664048 -0.437432 -1.980723
H -3.733370 4.051075 -0.986753
H -5.054252 3.860947 1.170365
I -1.396017 -1.052579 0.203770
I 1.304556 -1.302131 -0.867402
I -7.285221 -1.030455 -0.836086
I -9.978090 -1.507488 0.175683

Electronic Energy: -1186.1836668
Free Energy: -1186.083748

Compound: **Me-1I^{+}**

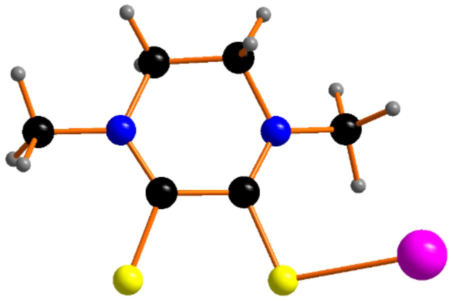

Cartesian Coordinates

S -0.542617 1.060353 -1.837127
N -0.281746 1.326130 -4.584080
C -0.341957 0.573691 -3.494523
C -0.586500 0.648849 -5.878291
H -1.671145 0.477694 -5.913303
H -0.301554 1.322180 -6.691730
C 0.005150 2.769267 -4.626308
H -0.920745 3.339320 -4.787110
H 0.689622 2.933635 -5.468363
H 0.478193 3.076725 -3.689565
S 0.080858 -1.638265 -2.073115
C -0.113379 -0.900050 -3.584420
N 0.052626 -1.478468 -4.775882
C 0.186678 -0.657638 -6.000867
H 1.260332 -0.481160 -6.172572
H -0.217311 -1.230675 -6.843231
C 0.441032 -2.895368 -4.874723
H -0.171649 -3.487155 -4.181883
H 1.502370 -3.011338 -4.607710
H 0.270150 -3.227992 -5.903735
I -2.111721 3.131755 -1.900422

Electronic Energy: -1151.5741535
Free Energy: -1151.456228

Compound: **Me-2**

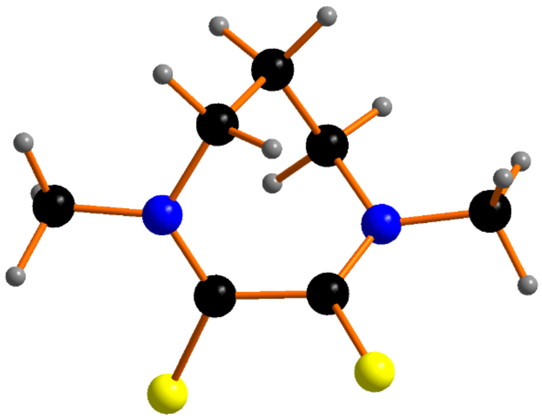

Cartesian Coordinates

S -3.536371 -1.387464 -2.170589
N -5.034918 -0.141205 -0.296613
C -3.900076 -0.792902 -0.639152
C -5.095483 0.588131 0.993575
C -4.178316 1.834954 0.987651
C -6.084172 0.109737 -1.295469
H -4.790731 -0.102266 1.791894
H -6.137500 0.870536 1.178076
H -4.062029 2.178373 2.026527
H -4.636411 2.651935 0.410230
H -5.739641 0.845448 -2.039831
H -6.973320 0.487497 -0.778666
H -6.321287 -0.824953 -1.819348
C -2.868042 -0.929949 0.469979
S -2.611523 -2.441066 1.164106
N -2.264247 0.221225 0.844335
C -2.800084 1.516512 0.359264
C -1.292036 0.231889 1.947198
H -2.885884 1.463543 -0.734659
H -2.071689 2.298799 0.598135
H -0.547206 -0.557691 1.785625
H -1.796655 0.039842 2.907681
H -0.802678 1.211877 1.970148

Electronic Energy: -1179.6034227
Free Energy: -1179.455358

Compound: **Me-2·I$_2$**

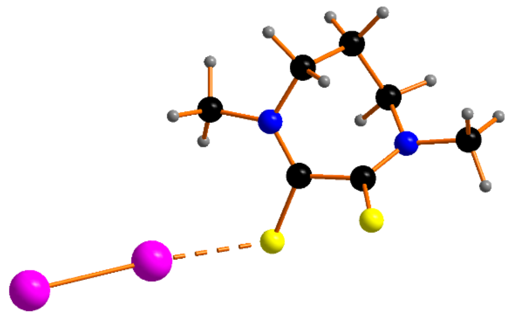

Cartesian Coordinates

S -3.504926 -1.055906 -2.360531
N -5.035385 -0.159649 -0.316254
C -3.869262 -0.667764 -0.771015
C -5.101089 0.440842 1.038798
C -4.294892 1.759484 1.110962
C -6.139835 0.103024 -1.252688
H -4.706052 -0.287627 1.759286
H -6.153874 0.612767 1.284962
H -4.171656 2.027321 2.170655
H -4.837399 2.577774 0.615911

H -5.892005 0.952929 -1.908110
H -7.041436 0.325347 -0.672031
H -6.303443 -0.782276 -1.880040
C -2.753763 -0.813112 0.255162
S -2.260610 -2.402901 0.648761
N -2.282875 0.321240 0.785215
C -2.918967 1.615013 0.417251
C -1.278190 0.362958 1.862008
H -3.027271 1.647646 -0.674182
H -2.237699 2.420060 0.711856
H -1.316246 -0.566997 2.439633
H -1.511430 1.218554 2.506997
H -0.273089 0.483689 1.434428
I 0.582362 -2.432850 0.467226
I 3.522932 -2.624465 0.118043

Electronic Energy: -1202.4720648
Free Energy: -1202.402013

Compound: **Me-2·$(I_2)_2$**

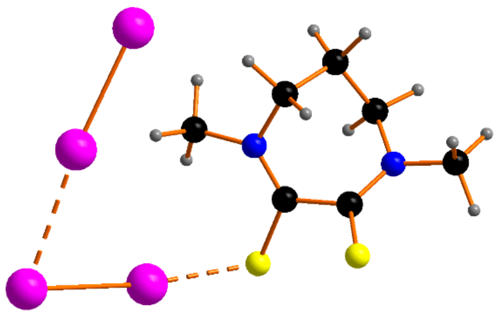

Cartesian Coordinates

S -3.508274 -1.270654 -2.242710
N -5.028264 -0.154159 -0.298649
C -3.891793 -0.767012 -0.692165
C -5.088969 0.526672 1.018779
C -4.198403 1.791913 1.041553
C -6.087545 0.127123 -1.281329
H -4.762301 -0.183554 1.789894
H -6.134216 0.779751 1.222617
H -4.084866 2.111658 2.087611
H -4.669439 2.614890 0.485570
H -5.766631 0.924181 -1.970327
H -6.988474 0.435293 -0.740549
H -6.289548 -0.778541 -1.867254
C -2.801513 -0.917215 0.356139
S -2.349803 -2.527763 0.800053
N -2.287427 0.204336 0.856443
C -2.819298 1.518334 0.397154
C -1.328791 0.261728 1.975807
H -2.894487 1.491843 -0.696866

H -2.084141 2.285848 0.662999
H -1.355942 -0.676455 2.538167
H -1.627674 1.099750 2.617879
H -0.317123 0.438489 1.587420
I 0.317220 -2.448422 0.397442
I 3.292676 -1.838711 -0.267962
I 2.141900 1.209192 -0.200309
I 0.772124 3.762282 -0.017103

Electronic Energy: -1225.4741447
Free Energy: -1225.344808

Compound: **Me-2·(I_2)(I_2)** on two sulphur atoms

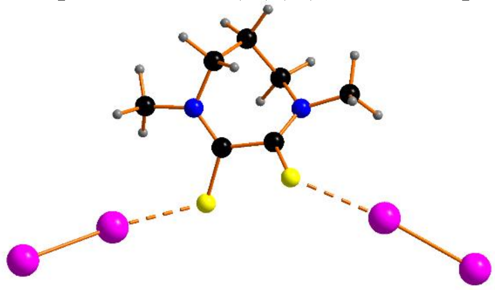

Cartesian Coordinates
S -3.518229 -1.239478 -2.397180
N -4.979767 -0.143133 -0.377715
C -3.843721 -0.703388 -0.814009
C -5.081350 0.354867 1.020244
C -4.317323 1.691310 1.162284
C -6.098103 0.203416 -1.273565
H -4.667127 -0.406028 1.692599
H -6.143533 0.471046 1.258559
H -4.195144 1.902590 2.234198
H -4.883882 2.521154 0.716262
H -5.720631 0.377119 -2.287290
H -6.571976 1.112260 -0.884140
H -6.830275 -0.615653 -1.294950
C -2.697717 -0.818063 0.190411
S -2.123558 -2.389756 0.506459
N -2.284734 0.315712 0.772509
C -2.947323 1.608040 0.450835
C -1.324202 0.338860 1.890616
H -3.066504 1.677682 -0.637074
H -2.279355 2.415452 0.767744
H -1.371658 -0.608124 2.439494
H -1.594241 1.173034 2.548974
H -0.304765 0.484863 1.507420
I 0.763935 -2.272886 0.449796
I 3.675803 -2.361176 0.169440
I -5.769974 -2.845898 -3.235934
I -7.852514 -4.700691 -4.123721

Electronic Energy: -1225.4720648
Free Energy: -1225.344885

Compound: **Me-2I$^+$**

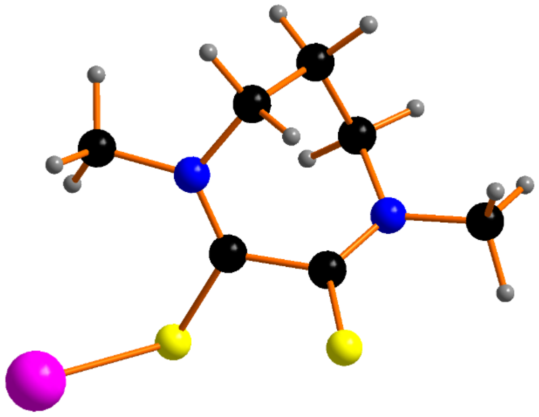

Cartesian Coordinates

S -1.286567 -2.184799 -0.918356
N -2.921761 -0.525541 0.484456
C -1.710488 -0.862006 0.010197
C -3.157880 0.849698 0.992286
C -3.124516 1.888368 -0.153683
C -4.082203 -1.390992 0.210694
H -2.389734 1.078240 1.742713
H -4.127887 0.861458 1.498640
H -3.045120 2.888612 0.294702
H -4.051979 1.853711 -0.741891
H -4.387850 -1.292241 -0.842292
H -4.900860 -1.089609 0.871799
H -3.806601 -2.435931 0.401626
C -0.588271 0.138770 0.198645
S 0.723264 -0.380972 1.242452
N -0.698283 1.321267 -0.387732
C -1.955178 1.631907 -1.135433
C 0.294900 2.406527 -0.273221
H -2.172368 0.781402 -1.793815
H -1.760107 2.508117 -1.761071
H 0.828475 2.323238 0.679026
H -0.248983 3.356222 -0.316651
H 1.004607 2.348311 -1.110265

S7. Radical trapping experiments

Experiments addressed isolating the possible products of the reaction at the solid state were carried out. The reaction was performed in acetone ($CHCl_3$, CH_2Cl_2, CH_3CN were avoided being reactive towards TEMPO) between Me-1:TEMPO:I_2 in the 1:1:1.5 molar ratio at room conditions. Within 3h,

the solution turned from dark brown to red, then after 24h it was found to be light red. No precipitation occurred during the 24h observation time. Slow evaporation of the solvent provided a precipitate that was recrystallized by Acetone/Et_2O obtaining a needle-shaped crystalline product that was characterized by FT-IR and Raman spectroscopy, elemental analysis, and single-crystal X-ray diffraction.

All the characterizations agreed on the formation of:

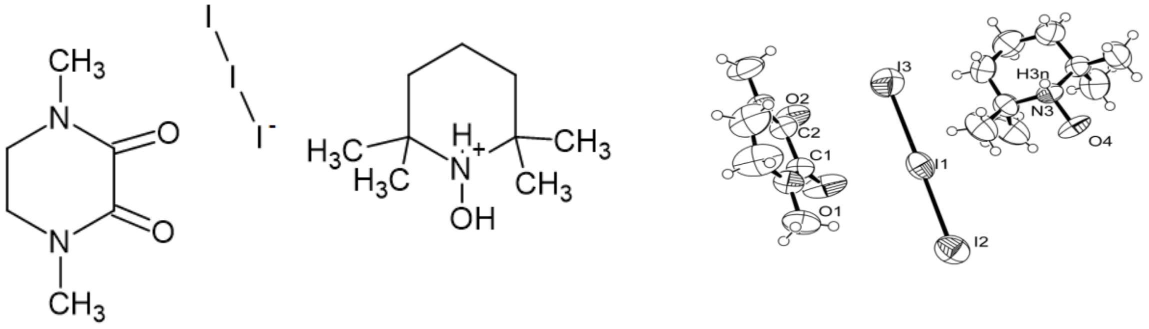

Figure S14. Not fully refined crystal structure of the product isolated from the **Me-1**+TEMPO+I_2 reaction in acetone.

as qualitatively shown by ORTEP (see Figure S14; refining was not satisfactory) and confirmed by elemental analysis (CHN-S: found C 26.62%, H 4.39%, N 6.24%, S -%; Calculated for $C_{15}H_{30}N_3O_3I_3$: C 26.45%, H 4.44%, N 6.17%). Furthermore, the Raman spectrum of the product showed the presence of the triiodide (ν(I-I-I) 111 cm^{-1}) and the FT-IR one showed the typical peaks expected for the N^+H-OH moiety, specifically ν(C-N$^+$) at 1388 cm^{-1}, ν(N$^+$-H) at 2859 and 2640-br cm^{-1}, ν(OH)/δ(OH) at 3152-br/1406 cm^{-1} (see Figure S15 and ref. S4, where IR frequencies of ν(N$^+$-H) were found at 2852 and 2620 cm^{-1}, ν(OH)/δ(OH) at 3080/1404 cm^{-1}).

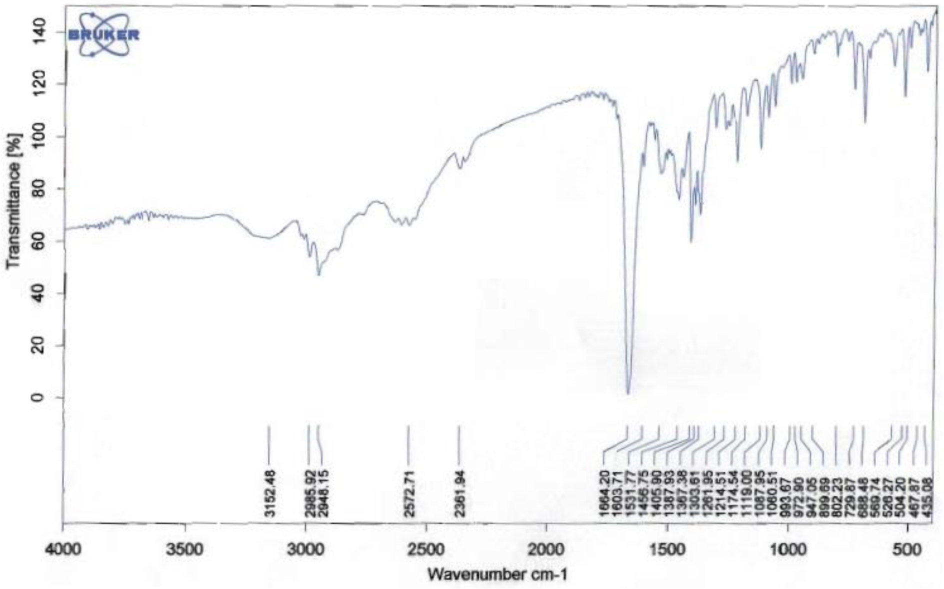

Figure S15. FT-IR spectrum of the product obtained from the **Me-1**+TEMPO+I_2 reaction in acetone.

Despite it was not possible to obtain a satisfactory refinement for the molecular structure of the recovered product, a radical reaction may be invoked for the formation of a species where **Me-1** underwent desulfurization on both the thionic groups and TEMPO is protonated.

References:

S1) Allen, F. H.; Watson, D. G.; Brammer, L.; Orpen, A. G.; Taylor, R. Typical Interatomic Distances: Organic Compounds. In *International Tables for Crystallography*; John Wiley & Sons, Ltd, **2006**; pp 790–811. https://doi.org/10.1107/97809553602060000621.

S2) De Ridder, D. J. A. Structure of N,N'-Dimethylpiperazine-2,3-Dithione: Space Group Correction. *Acta Cryst C* **1993**, *49*, 1975–1976. https://doi.org/10.1107/S0108270193005086.

S3) Perera, E.; Basu, P. Synthesis, Characterization and Structure of a Low Coordinate Desoxomolybdenum Cluster Stabilized by a Dithione Ligand. *Dalton Trans.* **2009**, *2009*, 5023–5028. https://doi.org/10.1039/B904113C.

S4) Percino, M.J.; Cerón, M.; Soriano-Moro, G.; Pacheco, J.A.; Castro, M.E.; Chapela, V.M.; Bonilla-Cruz, J.; Saldivar-Guerra, E., J. Mol. Struct. **2016**, *1103*, 254-264. http://dx.doi.org/10.1016/j.molstruc.2015.09.020.
